# Cyclic dipeptides from endophytic bacterium *Bacillus velezensis* as potential flavor precursors

**DOI:** 10.3389/fmicb.2025.1565502

**Published:** 2025-04-01

**Authors:** Tianxiao Li, Yaning Fang, Zhishun Chai, Lingbo Ji, Zhongrong Jiang, Dandan Meng, Baojiang He, Xiaolong Hu, Hui Xi, Xuewei Jia, Dongliang Li

**Affiliations:** ^1^Flavor Research Center, College of Food and Biological Engineering, Zhengzhou University of Light Industry, Zhengzhou, China; ^2^Cigar Fermentation Technology Key Laboratory of Tobacco Industry, China Tobacco Sichuan Industrial Co., Ltd., Chengdu, China; ^3^Zhengzhou Tobacco Research Institute of CNTC, Zhengzhou, China; ^4^China Tobacco Hunan Industrial Co., Ltd., Changsha, China

**Keywords:** cyclic dipeptide, *Bacillus velezensis*, flavor precursor, Py-GC-MS, flavor

## Abstract

Cyclic dipeptides represent a class of intriguing molecules with a wide range of biological activities, but their potential application as flavor precursors has not been previously reported. In this study, a flavor-producing bacterium *Bacillus velezensis* was screened out from 35 isolated endophytic bacteria. Gas chromatography-mass spectrometry (GC-MS) analysis suggested that the fermentation broth of *B. velezensis* contained flavor compounds and high amount of cyclic dipeptide flavor precursors. Three cyclic dipeptide flavor precursors, namely cyclo (L-prolyl-L-valine) (**1**), cyclo (L-prolyl-L-isoleucine) (**2**), and cyclo (L-prolyl-L-leucine) (**3**), were further isolated from the fermentation broth extraction through Sephadex LH-20 column chromatography and semi-preparative high-performance liquid chromatography (HPLC), and were identified by nuclear magnetic resonance (NMR) spectroscopy and mass spectrometry (MS). Flavor precursors can generate aroma components during pyrolysis, and the pyrolysis of compounds **1** and **3** were performed using pyrolysis GC-MS (Py-GC-MS) to analyze the flavor products. According to the relative odor activity value (ROAV) analysis, the key pyrolysis flavor compounds were revealed as 6-heptyl-5,6-dihydro-2*H*-pyran-2-one, isobutyric acid, 4-methyl-2-oxo-pentanoic acid ester, pyrrole derivatives, and pyrazine derivatives, which could give great contributions to milky, roasting, fruity, sweetness, and nutty aromas. The pyrolysis formation pathway of these flavor compounds was also proposed in detail. Addition of fermentation broth from the flavor-producing bacteria on cigar tobacco leaves significantly enhanced the milky, roasting, fruity, sweetness, and nutty aromas, which further demonstrated the flavor enhancing ability of cyclic dipeptides. This is the first report of flavor enhancing effects of cyclic dipeptides utilized as flavor precursors.

## Introduction

Flavor-producing microorganisms are extensively utilized in food production, including the fermentation of liquor, Huangjiu, vinegar, yoghurt, and sauce (Liu et al., 2022; Yan et al., 2022). This is attributed to the natural, economical, and distinctive flavors generated by these microbes. Various flavor components have been identified in the fermentation broths of these microorganisms, encompassing ketones, terpenes, alcohols, and aldehydes (Braga et al., 2018; Mindt et al., 2023). It has been reported that *Clostridium kluyveri* can synthesize flavor compound caproic acid in Chinese Baijiu (Yuan et al., 2022). Additionally, *Lactococcus* sp. and *Saccharomyces* sp. could enhance the yields of phenethyl alcohol during the Huangjiu fermentation process (Yan et al., 2022). The fungus *Talaromyces funiculosus* is capable of metabolizing sweet flavor compound 4-methyl-5,6-dihydropyran-2-one (Li et al., 2024a).

Cyclic dipeptides are a kind of interesting small molecules possessing 2,5-diketopiperazine scaffold, which can be formed through enzymatic catalysis or organic synthesis through condensation reaction followed by intramolecular cyclisation involving two α-amino acids (Großkopf et al., 2023; Ogilvie and Czekster, 2023). A diverse structures have been documented due to reactions between various types of α-amino acids and the highly stable feature (Kim and Cheng, 2024; Ogilvie and Czekster, 2023). Cyclic dipeptides exhibit a wide range of biological and pharmacological activities such as antibacterial, antifungal, antimalarial (Kim and Cheng, 2024; Liu et al., 2017; Pérez-Picaso et al., 2012). It has been reported that the cyclic dipeptides isolated from *Leuconostoc mesenteroides* LBP-K06 and endophytic fungus *Hormonema dematioides* exhibited significant antibacterial activities against multidrug-resistant bacteria *Staphylococcus aureus* 11,471, *Salmonella typhimurium* 12,219, and *S. aureus* (Kim and Cheng, 2024; Liu et al., 2017). Furthermore, eight cyclic dipeptides displayed antimalarial effects with IC_50_ values between 2.26 and 4.26 μM against *Plasmodium berghei* schizont (Pérez-Picaso et al., 2012). Moreover, several researches have revealed that cyclo (L-His-L-Pro) is a promising molecule for the treatment of neurological disorders such as Parkinson’s disease and schizophrenia, based on its neuronal maintaining effects (Li X. et al., 2024; Qian et al., 2020). Cyclic dipeptides are also recognized as ubiquitous signaling molecules in microbes communicate that facilitate communication to coordinate behavior through the secretion of small molecules (Ogilvie and Czekster, 2023).

Cyclic dipeptides represent an attractive class of compounds with diverse biological activities, but their application as flavor precursors has not been reported (Ogilvie and Czekster, 2023). Flavor precursors are defined as substances that possess little to no volatile aroma or exhibit only weak aromas themselves, but can generate flavors during processes such as aging, fermentation, combustion, and pyrolysis (Ayseli and Ayseli, 2016; Zang et al., 2022). Precursor compounds have been widely used in flavor and fragrance industry due to their high stability and durability (Li et al., 2021; Parker et al., 2017; Xiang et al., 2023). In this study, an endophytic flavor-producing bacterium, *B. velezensis*, was screened out through sensory evaluation, and its flavor compounds were identified by gas chromatography-mass spectrometry (GC-MS). The flavor precursor cyclic dipeptides were isolated from large scale fermentation broth of this bacterium using Sephadex LH-20 column chromatography (CC) and semi-preparative high-performance liquid chromatography (HPLC), and their structures were identified by MS and NMR data. Additionally, the key pyrolysis flavor products of cyclic dipeptides were determined by pyrolysis GC-MS (Py-GC-MS) and relative odor activity value (ROAV), and their possible formation pathways during pyrolysis were also speculated. The flavor enhancement effects on cigar tobacco leaves were further carried out and verified.

## Materials and methods

### General experimental procedures

GC-MS was performed using a 7890B-5977A GC-MS system (Agilent, United States). NMR data were recorded on Bruker AVIII-500 NMR instruments (Bruker, United States) with trimethylsilane (TMS) as the internal standard. Centrifugation was carried out using a TGL-16 M centrifuge (Shanghai Luxiangyi Centrifuge Instrument Co., Ltd.). UV–visible measurements were conducted using an Ultra-3400 spectrophotometer (Shenzhen Hualun Technology Co., Ltd.). The primary purification of secondary metabolites was carried out by CC using Sephadex LH-20 (Pharmacia, Sweden) as the parking material. The accurate purification was performed using Waters 1,525–2,998 semi-preparative HPLC (Waters, United States) that equipped with YMC-ODS-A HPLC column (250 × 10 mm, 5 μm, YMC, Japan). Py-GC-MS was performed through Shimadzu QP2020NX GC-MS in combination with thermal pyrolysis PY-3030D module. ESI-MS data were determined by a LTQ XL equipment (Thermo Fisher Technologies, United States).

### Chemicals and reagents

Dichloromethane, ethanol, hydrochloric acid, sodium hydroxide, sodium chloride, sodium hypochlorite solution, and anhydrous sodium sulfate were all analytical grade and obtained from Tianjing Fuyu Chemical Co., Ltd. (Tianjing, China). Standard flavor compounds such as 4-methyl-2-oxo-pentanoic acid ester, *N*-acetylpyrrolidone, and 2-pyrrolidinone were obtained from Sigma Co., Ltd. (Alexandria, United States). Yeast powder, agar powder, peptone, D-glucose were BR grade and obtained from Aoboxing Biotechnology Co., Ltd. (Beijing, China). Bacterial genomic DNA rapid extraction kit was obtained from Guangzhou Dongsheng Biotechnology Co., Ltd. (Guangzhou, China).

### Isolation of bacterial strains

The fresh tobacco leaves were collected at Qiubei county, Yunnan Province, China (24.13°N, 104.12°E) at July 2024, which were prepared into the small size of 1.0 cm^2^ in the laboratory. In order to completely remove the external microbial contamination on the leaf surface and obtain the endophytic bacterial strains, the tobacco leaf pieces were washed twice in 75% ethanol and disinfected using a 0.5% NaClO solution for 30 s. Then the leaf tissues were washed with sterile water twice and the edges were cut off. Each four sterilized leaf tissues were put on the surface of one Luria-Bertani (LB) medium plate, and incubated at 32°C for 48 h. The bacteria with different morphological structures on the LB medium were selected for further separation and purification. The number of bacterial colonies grown on the plate was recorded (Li et al., 2024a).

### Screening of flavor-producing bacteria

The isolated bacteria strains were cultured in a shaker at 32°C and 140 rpm for for 48 h. Subsequently, 2 mL of the liquid medium was centrifuged at 6,000 rpm for 5 min. The resulting supernatant was used for olfactory sensory evaluation. The sensory evaluation panel consisted of 12 professionally trained assessors. The main evaluation criteria included flavor intensity, irritation level, and retention time (Li et al., 2024a). Scores and aroma characteristics were meticulously recorded. Flavor intensity was rated on a scale of 0 to 20, with higher scores indicating stronger flavors. Irritation level and retention time were each rated on a scale of 0 to 10. Higher total scores indicated better flavor quality.

### Identification of bacterial strains

The bacterial strain with flavor-producing ability was selected for identification. The bacterium was cultivated on LB medium at 32°C for 48 h. The morphological characteristics on the surface of the colony were observed, and the cell microscopic features were observed under a microscope. The 16S rDNA of the obtained strain was extracted using rapid extraction kit, which was used as a template for PCR polymerase chain reaction. The PCR products was sent to Shanghai Sangon Biotech Co., Ltd. (Shanghai, China) for DNA sequencing. The results were blasted online in NCBI database. A phylogenetic tree was constructed using MEGA7 software through multiple comparison of the sequence. Neighbor-joining method was used with selected Kimura 2-parameter model, and the repeat time of Bootstrap was set to 1,000.

### GC-MS analysis of volatile compounds in the fermentation broth

The fermentation broths of the flavor-producing bacterium YUNM-8 and YUNM-12 were centrifuged, and the supernatant was extracted using equal volume of CH_2_Cl_2_ twice. The CH_2_Cl_2_ layers were combined and concentrated to 0.9 mL at 48°C, and 0.1 mL of phenylethyl acetate (0.8911 mg/mL) was added as an internal standard. Then the solution was filtered and used for GC-MS analysis.

GC-MS was performed using an Agilent 7890B-5977A GC-MS system that possessed with Agilent DB-5 MS column (30 m × 250 μm i.d. × 0.25 μm d.f.). The carrier gas was high purity helium (99.999%) with flow rate of 1.0 mL/min, and the injection volume was 1.0 μL. The column temperature was set as 50°C (held for 4 min) and increased to 240°C at 2°C/min. The MS ion source temperature was 230°C and quadrupole temperature was 150°C with ionization energy of 70 eV. Full scan mode was selected with mass scan range (*m*/*z*) of 35–550 amu.

For qualitative analysis, GC-MS data were preliminarily processed and compared with those in the NIST 20.0 spectral library, and compounds with matching scores greater than 85% were selected. Then, the retention indexes (RIs) was calculated according to the retention time of the n-alkane standards (C_6_–C_30_) under the identical GC-MS conditions. The RI value of each compounds was compared with those reported in the NIST Library database and the literatures (Li et al., 2024b). The key flavor compounds were further qualitatively identified by comparison of their retention times and ion fragments with those of the standard compounds in the authors’ laboratory.

For quantitative analysis, the internal standard method was used and phenylethyl acetate was used as the internal standard compound (0.8711 mg/mL).

### Isolation of flavor precursor compounds

The fermentation of *Bacillus velezensis* was performed in LB liquid broth (12 L) at 32°C for 48 h. The broth was centrifuged at 4,000 r/min for 10 min, and the supernatant was extracted with 1/3 volume of ethyl acetate three times. Then the ethyl acetate layers were concentrated using a rotary evaporation at 48°C to obtain a crude extract (262.8 mg).

The extract was dissolved in methanol and submitted to Sephadex LH-20 column chromatography. The mobile phase was CH_2_Cl_2_ and MeOH mixture. After combination, a total of 15 subfractions (S1–S15) were obtained and analyzed by GC-MS. The target part S9-S11 (120.6 mg) were combined and purified by semi-preparative HPLC. The mobile phase was MeOH:H_2_O = 35:65, and the flow rate was 4.0 mL/min. The peaks at retention time of 24.9 min, 35.3 min, and 36.2 min were collected and evaporated to obtain compound **1** (white solid, 5.3 mg), **2** (white solid, 1.6 mg), and compound **3** (white solid, 6.1 mg). The isolated compounds **1**–**3** were dissolved in CD_3_OD with trimethylsilane (TMS) as the internal standard for NMR measurement. The MS data were also measured using their methanol solution.

### Py-GC-MS analysis of precursor compounds

Py-GC-MS was carried out with pyrolysis atmosphere of 10% oxygen and 90% nitrogen (Zang et al., 2022). The temperature of cracker accessories and transmission line was both 250°C. The temperature rise program was initial temperature of 50°C with heating rate 20°C/ms to the final temperature of 600°C, which was held for 10 s. The GC-MS column was Agilent HP-5 MS chromatographic column (30 m × 250 μm i.d. × 0.25 μm d.f.). The column temperature was set as 50°C (held for 2 min), increased to 150°C at 3°C/min (held for 5 min), and then increased to 280°C at 8°C/min. The other conditions were the same as above.

### Olfactory threshold and ROAV determination

The olfactory threshold of each pyrolysis flavor compound was determined through the best estimate threshold (BET) method in water (Li et al., 2024a; Niu et al., 2018). The flavor compounds were prepared to a concentration of 0.5 mg/g. After nine serial dilutions, a series of solutions were obtained. Sensory evaluations were performed by a panel of 12 trained evaluators (six males and six females, aged 22–30 years) with over 4 years of sensory experience. Prior to the formal experiment, the panelists completed an intensive 30-day training program, consisting of daily 2-h sessions. The training was designed to increase their proficiency in evaluating aroma descriptions and aroma intensities of diverse aromatic compounds. A three-point selection test was performed by the evaluators for each concentration. For each flavor compound, the estimation threshold was determined using the geometric mean of the highest concentration at which each evaluator identified the compound and its adjacent higher concentration. The geometric mean of the BET values from 12 evaluators was calculated as the olfactory threshold. The contribution of each flavor compound to the overall aroma was evaluated by its odor activity value (OAV), which was defined in this manuscript as OAV = relative content/detection threshold. To further compare the contribution of each flavor compound, the ROAV was calculated using the flavor compound with highest OAV as standard (ROAV = 100).

### Flavor enhancement on cigar tobacco leaves and sensory evaluation verification

The strains *B. velezensis* and *S. equorum* were cultivated in LB liquid medium for 24 h. Then the cultures was centrifuged at 6,000 r/min for 10 min and the supernatant was taken out. Subsequently, 2.5 mL of supernatants together with 11.5 mL of deionized water were uniformly sprayed on the surface of Dexue No. 1 cigar tobacco leaves (100 g). The control groups were sprayed with 14.0 mL of deionized water. The tobacco leaves were kept at 22°C and 60% relative humidity for 8 h. Subsequently, the tobacco leaves were rolled into 44 rings cigars (diameter, about 1.0 cm; circumference, about 3.1 cm) for sensory evaluation.

The sensory evaluation panel consisted of 12 professionally trained assessors as above mentioned. Each assessor was given nine cigars (three control group cigars, three *B. velezensis* fermentation broth addition cigars, and three *S. equorum* fermentation broth addition cigars). Following the standardized sensory quality evaluation method for cigars, the main aromas for sensory evaluation were selected as roasting, fresh sweetness, milky, fruity, floral, and nutty, with scores ranging from 0 to 5 (Hu et al., 2022; Jiang et al., 2024). Scores of 4–5 indicated a very strong intensity, while 3–4 denoted strong intensity. Scores of 1–3 represented moderate intensity, and 0–1 was classified as weak. The average sensory score of each aroma was recorded by every assessor on the basis of the triplet repetition cigar samples. Finally, the sensory scores of 12 assessors were summarized, and the average sensory scores were calculated. The data were shown in mean ± SD values.

## Results and discussion

### Bacterial strain isolation and screening

Thirty-five strains of endophytic bacteria were isolated from fresh tobacco leaves and designated as YUNM-1 to YUNM-35. The liquid fermentation broth of all 35 bacteria strains were screened for flavor-producing. Notably, liquid fermentation broth of five bacterial strains namely YUNM-2, YUNM-7, YUNM-8, YUNM-12, and YUNM-17 exhibited obvious and rich flavors (Table 1). And the fermentation broth of YUNM-12 obtained the highest score, which was selected for further research.

**Table 1:** Olfactory sensory evaluation results of flavor-producing bacteria.

Strain	Flavor strength	Irritation level	Retention time	Total score
YUNM-2	17.6	7.8	7.6	33.0
YUNM-7	17.8	8.1	8.2	34.1
YUNM-8	17.9	8.0	8.5	34.4
YUNM-12	18.2	8.9	9.0	36.1
YUNM-17	17.4	7.3	7.4	32.1

### Bacterial strain identification

**Figure 1:**
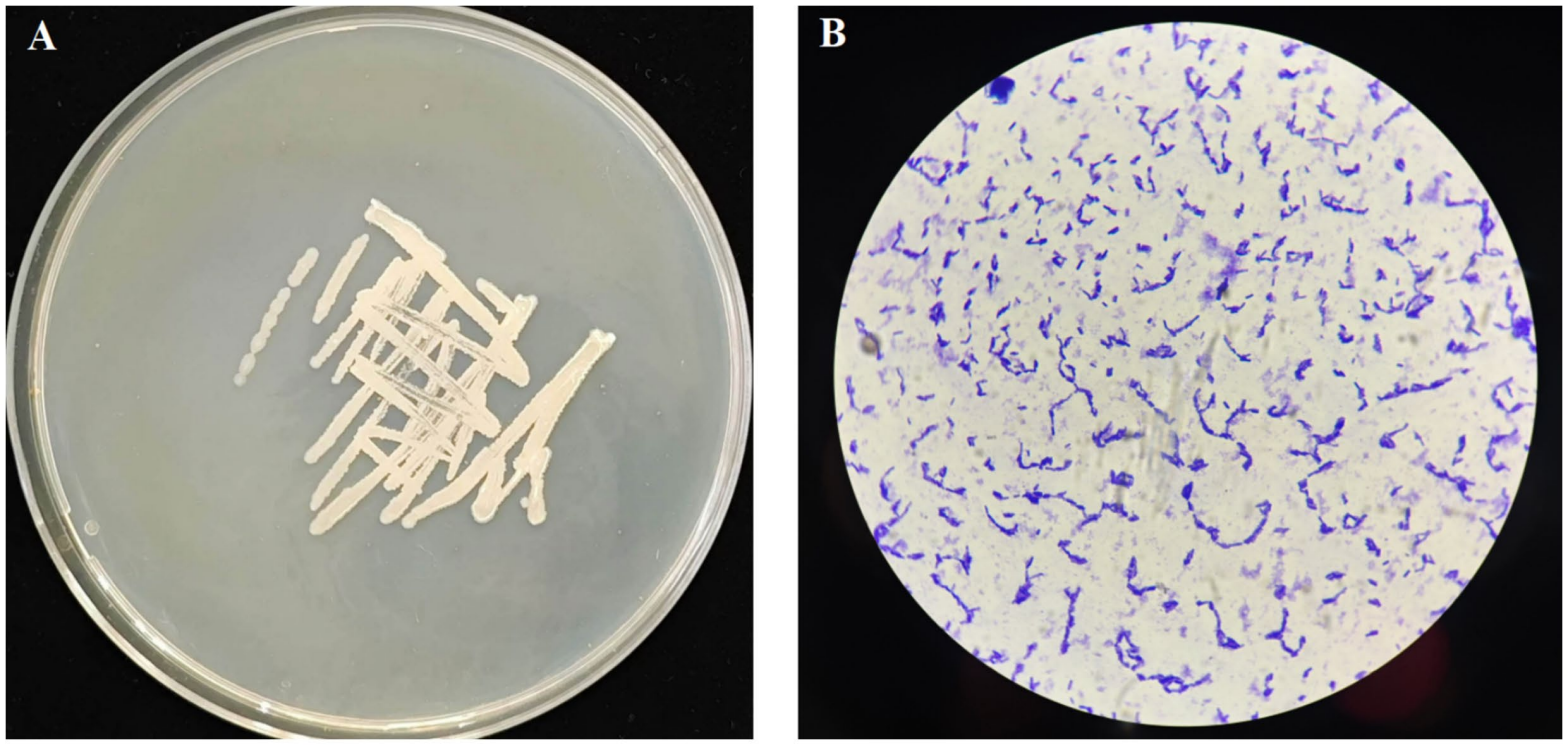
Morphological (**A**, LB medium) and microscopic characteristics (**B**, 10 × 100) of YUNM-12.

**Figure 2:**
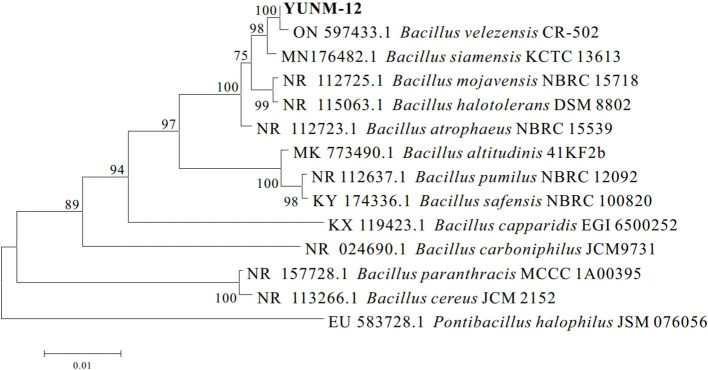
Phylogenetic tree of *B. velezensis* YUNM-12.

The YUNM-12 strain was cultured on the LB agar plate for 24 h, and the morphological characteristics were shown in Figure 1. The colony was white with smooth edges, and fusiform bacteria cell could be observed under a microscope. The bacterial 16S rDNA sequence was 100% identity to those of *Bacillus velezensis* strains in the NCBI database, particularly matching *B. velezensis* ON597433.1. A phylogenetic tree was constructed, indicating that bacterial strain YUNM-12 belonged to the same branch as *B. velezensis* (Figure 2). Therefore, based on its colony morphology, microscopic characteristics, and 16S rDNA sequence analysis, YUNM-12 strain was identified as *B. velezensis*.

### GC-MS analysis of volatile compounds in fermentation broth of *Bacillus velezensis* YUNM-12

As shown in Table 2, a total of 32 volatile compounds were identified in the fermentation broth of *B. velezensis*. The concentrations of 1-phenylethyl acetate and phenethyl butyrate were determined at levels of 1.61 μg/mL and 1.30 μg/mL, respectively. The two compounds exhibited floral, fruity, and sweet aroma. The terpenes such as 1-pentadecene, 1-eicosene, and (*Z*)-3-heptadiene also contributed to floral flavor.

**Table 2:** GC-MS analysis of main aroma components in fermentation broth of *B. velezensis*.

No.	*t*_R_ (min)	*RI*/(cal./ref.)	Qualitative	Flavor compounds	Concentration (μg/mL)	
					LB blank medium	Fermentation broth of *B. velezensis*
1	10.90	1,192/1,194	RI, MS, S	1-Phenylethyl acetate	—	1.61
2	11.73	—	MS	2-Ethylbutyl isobutyrate	—	1.59
3	15.69	1,180/1,182	RI, MS, S	Octanoic acid	0.29	—
4	17.64	1,200/1,200	RI, MS, S	Dodecane	0.18	0.39
5	20.39	1,400/1,400	RI, MS, S	Tetradecane		0.19
6	24.30	1,443/1,444	RI, MS, S	Phenylethyl butyrate	0.03	1.30
7	27.15	1,493/1,492	RI, MS, S	1-Pentadecene	—	0.11
8	27.72	1,504/1,505	RI, MS, S	2,6-Bis(1,1-dimethylethyl)-4-methyl-phenol	—	0.07
9	27.88	1,514/1,513	RI, MS, S	2,4-Bis(1,1-dimethylethyl)-phenol	—	0.12
10	31.78	—	MS	Undecylcyclopentane	—	0.16
11	32.86	—	MS	(*Z*)-3-Heptadiene	—	0.14
12	34.11	—	MS	Cyclo(L-prolyl-L-alanine)	—	6.05
13	34.30	—	MS	3-Methyl-6-(1-methylpropyl)-2,5-piperazinedione	—	0.67
14	34.91	—	MS	Cyclo(L-prolyl-L-glycine)	—	9.82
15	36.07	1,802/1,803	RI, MS, S	Cyclo(L-prolyl-L-valine)	1.18	7.15
16	37.19	—	MS	(1-Methylethyl)-cycloundecane	—	0.26
17	38.45	—	MS	Cyclo(L-prolyl-L-isoleucine)	—	6.36
18	38.96	1,907/1,908	RI, MS, S	Cyclo(L-prolyl-L-leucine)	—	27.69
19	39.12	1,964/1,963	RI, MS, S	Hexadecanoic acid	0.17	—
20	39.65	1,971/1,970	RI, MS, S	Dibutyl phthalate	—	0.37
21	40.70	—	MS	5,5-Diethylheptadecane	—	0.08
22	42.10	1,991/1,994	RI, MS, S	1-Eicosene	—	0.23
23	42.74	—	MS	(*Z*)-5-Nonene	—	0.19
24	42.89	2,100/2,100	RI, MS, S	Heneicosane	—	0.05
25	44.48	—	MS	Octadecylcyclohexane	—	0.29
26	46.59	2,191/2,192	RI, MS, S	1-Docosadiene	—	0.36
27	48.62	—	MS	Cyclo(L-prolyl-L-phenylalanine)	—	10.71
28	49.18	2,399/2,398	RI, MS, S	Hexanedioic acid Bis(2-ethylhexyl) ester	—	0.58
29	50.47	2,400/2,400	RI, MS, S	Tetracosane	—	0.23
30	52.04	2,520/2,519	RI, MS, S	Bis(2-ethylhexyl) phthalate	5.88	20.34
31	52.37	—	MS	2-Methyl-6-[4-(4-methylpentyl)cyclohexyl]heptane	—	0.18
32	55.72	—	MS	Dioctyl terephthalate	—	0.23

Moreover, several cyclic dipeptides such as cyclo (L-prolyl-L-alanine), cyclo (L-prolyl-L-glycine), cyclo (L-prolyl-L-valine), cyclo (L-prolyl-L-isoleucine), cyclo (L-prolyl-L-leucine), and cyclo (L-prolyl-L-phenylalanine) could be identified in the fermentation broth with the concentrations from 6.05 to 27.69 μg/mL. Only cyclo (L-prolyl-L-valine) with low concentration was identified from LB blank medium.

To further verify the flavor-producing abilities of the isolated endophytic bacteria, another strain YUNM-8 that identified as *Staphylococcus equorum* was cultivated in LB medium, and the flavor compounds in the fermentation broth were also determined by GC-MS (Supplementary Table S1). Four cyclic dipeptides including cyclo (L-prolyl-L-glycine) (19.31 μg/mL), cyclo (L-prolyl-L-valine) (7.80 μg/mL), cyclo (L-prolyl-L-leucine) (14.41 μg/mL), and cyclo (L-prolyl-L-phenylalanine) (8.10 μg/mL) were also identified in the fermentation broth. These cyclic dipeptides were considered as secondary metabolites of flavor-producing bacteria, but their flavor enhancing effects have not been reported. Since the numbers and concentrations of cyclic dipeptide precursor compounds in the fermentation broth of *B. velezensis* were larger and higher, a large scale fermentation using this strain was further performed.

### Structural elucidation

**Figure 3:**
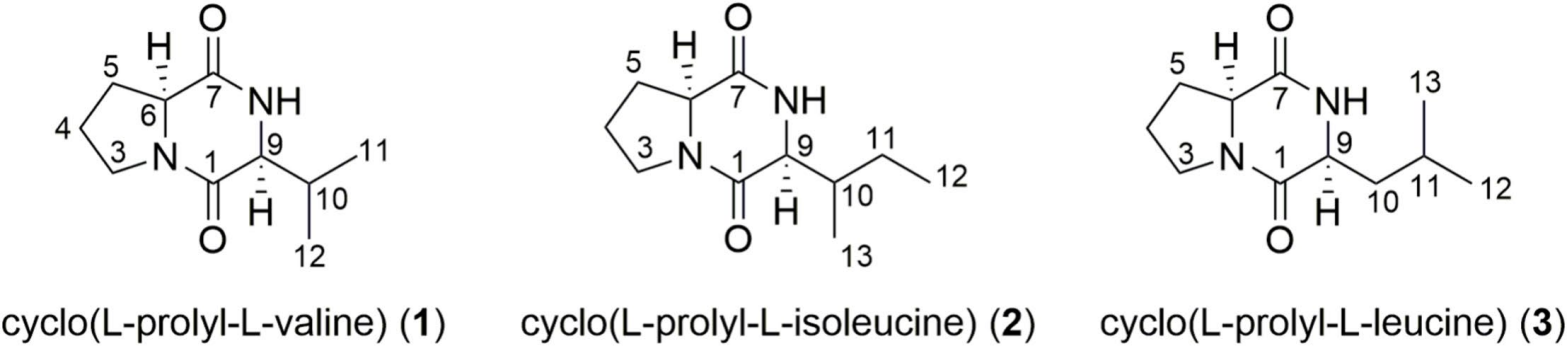
Structures of cyclo (L-prolyl-L-valine) (**1**), cyclo (L-prolyl-L-isoleucine) (**2**), and cyclo (L-prolyl-L-leucine) (**3**).

The liquid fermentation broth (12 L) of *B. velezensis* was extracted with EtOAc to acquire a crude extract, which was further purified by Sephadex LH-20 and semi-preparative HPLC to yield three metabolites cyclo (L-prolyl-L-valine) (**1**), cyclo (L-prolyl-L-isoleucine) (**2**), and cyclo (L-prolyl-L-leucine) (**3**) (Figure 3). The isolated compounds **1**–**3** were dissolved in CD_3_OD with trimethylsilane (TMS) as the internal standard for NMR measurements. The MS data were also recorded using their methanol solution.

Compound **1** was obtained as white powder. Its molecular formula C_10_H_16_N_2_O_2_ was established from positive ESI-MS [M + H]^+^ ion at *m*/*z* 197.06 (Supplementary Figure S1) in combination with its ^1^H NMR and ^13^C NMR data (Supplementary Table S2). The ^1^H NMR data (Supplementary Figure S2) clearly indicated the presence of three methines including two connected with nitrogen atom (*δ*_H_ 4.20, 1H, t, *J* = 7.3 Hz, H-6; 4.03, s, H-9; 1.95, m, H-10), three methenes including one connected with nitrogen atom (*δ*_H_ 3.56, 3.50, each 1H, m, H-3; 2.48, 2.32, each 1H, m, H-5; 2.02, 1.95, each 1H, m, H-4), and two methyls (*δ*_H_ 1.09, 3H, d, *J* = 7.3 Hz, H_3_-11; 0.94, 3H, d, *J* = 6.9 Hz, H_3_-12). A total of 10 carbon signals could be disclosed from its ^13^C NMR spectrum (Supplementary Figure S3), which could be classified as two amide carbonyls (*δ*_C_ 173.0, C-7; 167.7, C-1), three methines including two connected with nitrogen atom (*δ*_C_ 61.7, C-6; 60.2, C-9; 29.4, C-10), three methenes including one connected with nitrogen atom (*δ*_C_ 46.3, C-3; 30.1, C-5; 23.4, C-4), and two methyls (*δ*_C_ 19.0, C-11; 16.8, C-12). The NMR data were in accordance with those in the literatures (Fdhila et al., 2003; Schmidtz et al., 1983). Thus, the structure of compound **1** was determined as cyclo (L-prolyl-L-valine).

The positive ESI-MS [M + H]^+^ ion of **2** at *m*/*z* 211.09 (Supplementary Figure S4) suggested molecular weight of 210, which gave molecular formula C_11_H_18_N_2_O_2_ for **2** in combination with its ^1^H NMR and ^13^C NMR data (Supplementary Table S2). The NMR data were highly similar with those of **1**, except for the presence of one more methene (*δ*_H_ 1.43, 1.32, each 1H, m, H-11; *δ*_C_ 23.3, C-11), a triplet methyl (*δ*_H_ 0.93, 3H, t, *J* = 7.4 Hz, H_3_-12; *δ*_C_ 12.7, C-12), a changed methine (*δ*_H_ 2.16, 1H, m, H-10; *δ*_C_ 37.2, C-10), and a changed doublet methyl (*δ*_H_ 1.07, 3H, d, *J* = 7.1 Hz, H_3_-13; *δ*_C_ 15.7, C-13) (Supplementary Figures S5, S6). Thus, the isopropyl side chain of **1** was replaced as a sec-butyl group in **2**. Moreover, the NMR data were in accordance with those in the literatures (Fdhila et al., 2003; Schmidtz et al., 1983) and the structure of compound **2** was confirmed as cyclo (L-prolyl-L-isoleucine).

Compound **3** was revealed as an isomer of **2** due to the same molecular formula C_11_H_18_N_2_O_2_, which was demonstrated from positive ESI-MS [M + H]^+^ ion at *m*/*z* 211.11 (Supplementary Figure S7) and the NMR data (Supplementary Table S2). The side chain of **3** was assigned as an isobutyl group from the NMR signals of a methine (*δ*_H_ 1.51, 1H, m, H-11; *δ*_C_ 25.9, C-11), a methene (*δ*_H_ 1.90, 2H, m, H-10; *δ*_C_ 39.6, C-10), and two doublet methyl (*δ*_H_ 0.97, 3H, d, *J* = 6.0 Hz, H_3_-12 and 0.95, 3H, d, *J* = 6.0 Hz, H_3_-13; *δ*_C_ 23.4, C-12 and 22.4, C-13) (Supplementary Figures S8, S9). The NMR data of **3** were in accordance with those in the literatures (Fdhila et al., 2003; Schmidtz et al., 1983), and its structure was elucidated as cyclo (L-prolyl-L-leucine).

### Py-GC-MS analysis of compound **1**

As shown in Table 3, overall 22 pyrolysis products derived from cyclo (L-prolyl-L-valine) were found. Apart from the compound itself, the products with high amount were revealed as 1,5-dihydro-4-(1-pyrrolidinyl)-2*H*-pyrrol-2-one, 6-heptyl-5,6-dihydro-2*H*-pyran-2-one, and 1,4-butanediol. The compound 6-heptyl-5,6-dihydro-2*H*-pyran-2-one is recognized as a typical lactone that associate with milky, sweetness, and cream flavor (Zang et al., 2022; Li et al., 2024a). 1-Hexanol and myristic acid also possess sweetness and milky flavor, while isobutyric acid contributes to the flavor of yogurt and fruit. Moreover, compounds such as 1,5-dihydro-4-(1-pyrrolidinyl)-2*H*-pyrrol-2-one, 2-pyrrolidinone, 1-piperidino-1-butanone, 2-(1*H*-pyrrol-2-yl)-piperidine, and 2,5-dimethyl-3-isopropylpyrazine are contributed to roasting and nutty aromas.

**Table 3:** Main pyrolysis products of compound **1** at 600°C.

No.	*t*_R_ (min)	Flavor compounds	Peak area/%
1	1.46	1,4-Butanediol	0.19
2	1.60	1-Hexanol	0.16
3	1.91	2-Methyl-1-hexene	0.01
4	2.34	Isobutyronitrile	0.05
5	3.13	Isobutyric acid	0.02
6	5.77	2,4-Dimethyl-3-pentanone	0.01
7	7.89	2-Pyrrolidinone	0.03
8	9.09	Glycyl-DL-leucine	0.01
9	9.39	Dodecane	0.01
10	9.53	Decanal	0.01
11	10.46	1-Piperidino-1-butanone	0.13
12	10.46	Hydroxyproline	0.12
13	11.62	1-Oxaspiro[4.4]nonan-4-one	0.01
14	11.87	1,1’-Carbonylbis-2-pyrrolidinone	0.08
15	12.36	2,5-Dimethyl-3-isopropylpyrazine	0.02
16	12.57	6-Heptyl-5,6-dihydro-2*H*-pyran-2-one	0.26
17	14.37	Androsta-1,4,6-triene-3,17-dione	0.17
18	15.03	5-Methyl-cytidine	0.01
19	15.46	Myristic acid	0.02
20	16.87	2-(1*H*-pyrrol-2-yl)-piperidine	0.01
21	17.37	Cyclo(L-prolyl-L-valine)	68.14
22	18.59	1,5-Dihydro-4-(1-pyrrolidinyl)-2*H*-pyrrol-2-one	12.77

The contribution of each volatile compound to the overall aroma can be evaluated by its ROAV. A volatile compound is distinguished as key flavor compound when its ROAV ≥1.00, while 0.1 ≤ROAV <1.00 suggests it could be classified as modification component. As shown in Table 4, the ROAV of seven main pyrolysis products of **1** at 600°C was calculated. Interestingly, the contribution of 6-heptyl-5,6-dihydro-2*H*-pyran-2-one to the overall aroma was particularly prominent and it was selected as standard compound, since its olfactory threshold is low (0.0037 μg/g). 2,5-Dimethyl-3-isopropylpyrazine, 1-hexanol, and isobutyric acid were also revealed as key flavor compounds with great contributions (ROAV ≥1.00), while decanal could be assigned as modification component. Overall, these compounds mainly contributed to milky, sweetness, roasting, nutty, and fruity aromas.

**Table 4:** Olfactory thresholds and ROAVs of main pyrolysis products of **1**.

Compounds	*T*_ref._ (μg/g)	*T*_test._ (μg/g)	Odor	Relative content	OAV	ROAV
1-Hexanol	0.0056	0.074	Fruity, milky, sweetness, floral	0.16	2.16	3.08
Isobutyric acid	0.01	0.01	Fruity, yogurt	0.02	2.00	2.85
2-Pyrrolidinone	—	1.07	Roasting, sweetness, milky	0.03	0.03	0.04
Decanal	0.0001	0.046	Citrus, freshness, sweetness, floral	0.01	0.22	0.31
2,5-Dimethyl-3-isopropylpyrazine	—	0.0023	Nutty, roasting, fruity	0.02	8.70	12.37
6-Heptyl-5,6-dihydro-2*H*-pyran-2-one	0.00005	0.0037	Milky, creamy, sweetness	0.26	70.27	100.00
Myristic acid	1.00	1.00	Milky, coconut, creamy	0.02	0.02	0.03

**Figure 4:**
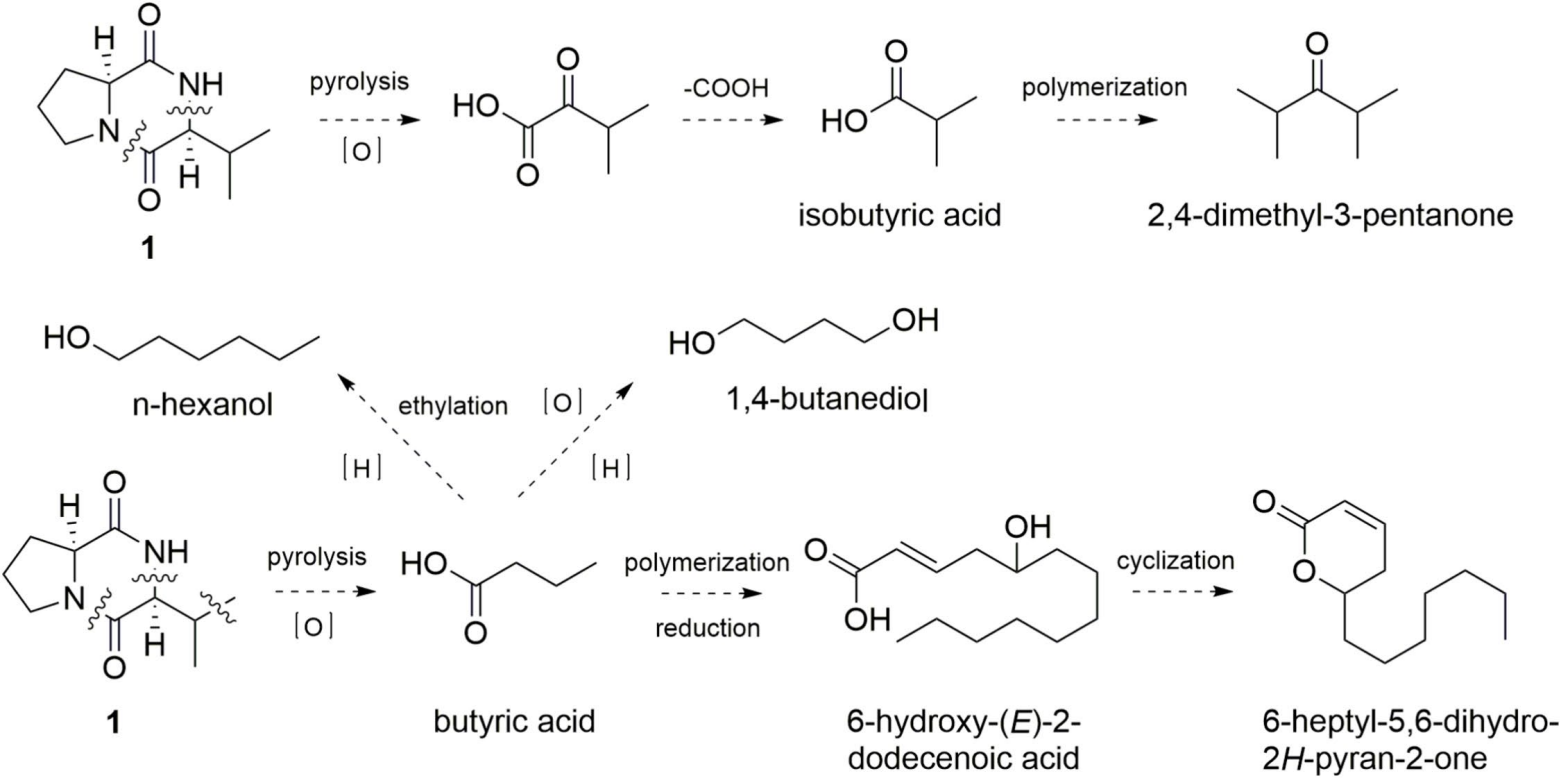
Possible formation pathway of acid and lactone flavor compounds through pyrolysis of **1**.

As shown in Figure 4, the simultaneously broken of 1,2-amide bond and 8,9-C–N bond of **1** would bring an intermediate, which was further oxidized to form 3-methyl-2-oxo-butyric acid and decarboxylated to yield isobutyric acid. Polymerization and decarboxylation of isobutyric acid gave 2,4-dimethyl-3-pentanone. In the same manner, broken of 1,2-amide bond, 8,9-C–N bond, and 10,11-C–C bond of **1** would obtain butyric acid, which was further oxidized and reduced to form 1,4-butanediol. Butyric acid reacted with ethyl radical to yield n-hexanol. Moreover, polymerization, reduction, dehydration of three butyric acid could obtain the key intermediate compound 6-hydroxy-(*E*)-2-dodecenoic acid, which could form the key sweetness and milky aroma compound 6-heptyl-5,6-dihydro-2*H*-pyran-2-one through esterification and cyclization.

**Figure 5:**
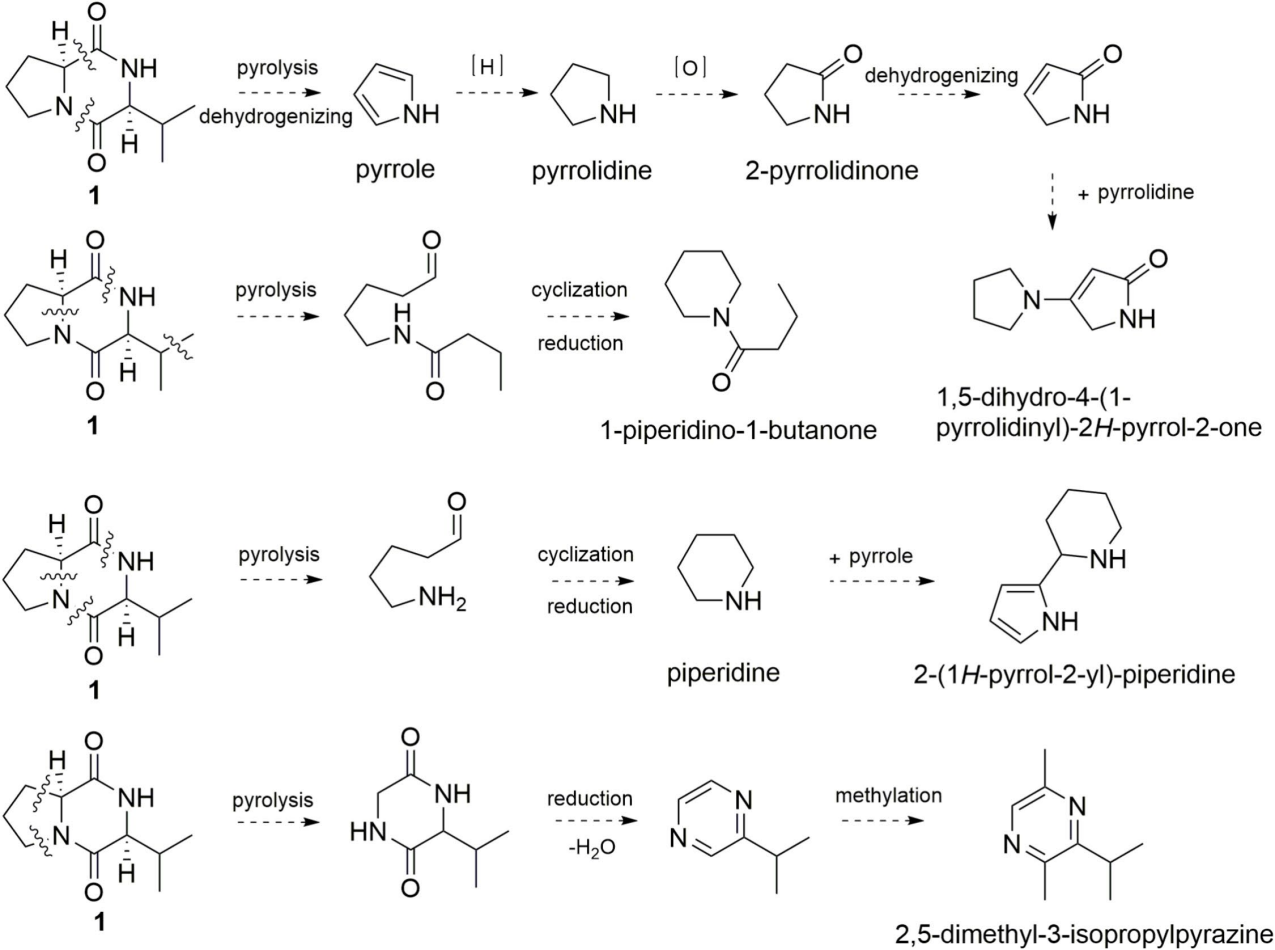
Possible formation pathway of pyrrole and pyrazine flavor compounds through pyrolysis of **1**.

On the other hand, the simultaneously broken of 1,2-amide bond and 6,7-C–C bond of **1** would yield pyrrole, pyrrolidine, 2-pyrrolidinone, and 1,5-dihydro-4-(1-pyrrolidinyl)-2*H*-pyrrol-2-one (Figure 5). 1-Piperidino-1-butanone and 2-(1*H*-pyrrol-2-yl)-piperidine could be formed in the same manner. Broken of 2,3-C-N bond and 5,6-C–C bond of **1** would yield isopropyl diketopiperazine and 3-isopropyl pyrazine, which reacted with methyl radical to form 2,5-dimethyl-3-isopropylpyrazine. Thus, pyrolysis product pyrrole is mainly derived from the five-membered ring of proline, and pyrazine is generated from the six-membered ring of diketopiperazine.

### Py-GC-MS analysis of compound **3**

Interestingly, more flavor components were found in pyrolysis products of cyclo (L-prolyl-L-leucine) (**3**) than those of **1** (Table 5). Apart from compound **3** itself, 1,5-dihydro-4-(1-pyrrolidinyl)-2*H*-pyrrol-2-one, 2-ethyl-3-methylpyrazine, and 2-methylpyrrole were assigned as the main products with high amount. Several compounds such as 4-methyl-2-oxo-pentanoic acid, 4-methyl-2-oxo-pentanoic acid methyl ester, 3-methyl-butanoic acid, myristic acid, and 2-methyl-2-cyclopenten-1-one could be related to milky and fruity flavor (Zang et al., 2022; Li et al., 2024a, 2024b). 4-Methyl-2-oxo-pentanoic acid, 4-methyl-2-oxo-pentanoic acid methyl ester, and 3-methyl-butanoic acid possess fruity and yogurt flavor. 2-Methyl-2-cyclopenten-1-one is a kind of typical caramel and milky flavor compounds. Myristic acid is revealed as the characteristic aroma compound of coconut and ice-cream. Moreover, 1,5-dihydro-4-(1-pyrrolidinyl)-2*H*-pyrrol-2-one, 2-ethyl-3-methylpyrazine, 2-methylpyrrole, 3-methylpyrrole, pyrrole, 2,4-dimethylpyrrole, 2-pyrrolidone, and 2-acetyl-1-pyrroline are considered as typical roasting and nutty aroma components.

**Table 5:** Main pyrolysis products of compound **3** at 600°C.

No.	*t*_R_ (min)	Flavor compounds	Peak area/%
1	3.02	Pyrrole	0.01
2	3.13	2-Methyl-2-cyclopenten-1-one	0.02
3	4.12	2-Methylpyrrole	0.12
4	4.18	3-Methylpyrrole	0.01
5	4.22	3-Methyl-butanoic acid	0.04
6	4.75	Styrene	0.01
7	5.16	2-Acetyl-1-pyrroline	0.01
8	5.39	2,4-Dimethylpyrrole	0.01
9	6.45	3-Methyl-butanamide	0.09
10	6.76	2,6-Dimethyl-7-octen-2-ol	0.01
11	7.04	3-Methyl-*N*-(3-methylbutylidene)-1-butanamine	0.04
12	7.36	4-Methyl-2-oxo-pentanoic acid methyl ester	0.08
13	7.42	4-Methyl-2-oxovaleric acid	0.08
14	7.78	2-Pyrrolidinone	0.12
15	8.60	(*Z*)-2-Methyl-2-butenediamide	0.03
16	8.86	Undecanenitrile	0.07
17	9.19	Cyclopentanone oxime	0.01
18	10.88	2-Methylpyrrolidine	0.03
19	11.87	*N*-acetylpyrrolidone	0.20
20	13.50	2-Ethyl-3-methyl-pyrazine	0.15
21	14.42	2-(1-Methylethylidene)-cyclohexanone	0.15
22	14.75	1,2-Dihydro-3-isobutyl-1-methyl-2-pyrazinone	0.10
23	14.86	2,4-Dimethyl-hexane	0.13
24	16.38	Myristic acid	0.04
25	17.37	Vinylcyclohexyl ether	0.40
26	18.52	Cyclo(L-prolyl-L-leucine)	57.46
28	20.56	1,5-Dihydro-4-(1-pyrrolidinyl)-2*H*-pyrrol-2-one	17.33

The ROAV was calculated and shown in Table 6. 2,5-Dimethyl-3-isopropylpyrazine was selected as standard compound that brought significant contribution to the aroma, since its OAV was largest (85.23). 2-Acetyl-1-pyrroline, 2-methylpyrrole, 3-methyl-butanoic acid, 4-methyl-2-oxo-pentanoic acid methyl ester, and *N*-acetylpyrrolidone could also be distinguished as key flavor compounds with great contributions to the overall aroma (ROAV ≥1.00). Styrene, 2,4-dimethylpyrrole, 4-methyl-2-oxovaleric acid, and 2-pyrrolidinone could be assigned as modification components. Overall, the flavor of pyrolysis compounds of **3** was more rich than those of **1**, possibly due to the larger number of main flavor compounds.

**Table 6:** Olfactory thresholds and ROAVs of main pyrolysis products of **3**.

Compounds	*T*_ref._ (μg/g)	*T*_test._ (μg/g)	Odor	Relative content	OAV	ROAV
Pyrrole	—	1.00	Roasting, nutty	0.01	0.01	0.01
2-Methyl-2-cyclopenten-1-one	0.30	0.30	Caramel, milky	0.02	0.07	0.08
2-Methylpyrrole	—	0.097	Roasting, nutty	0.12	1.24	1.45
3-Methylpyrrole	—	0.15	Roasting, nutty	0.01	0.07	0.08
3-Methyl-butanoic acid	0.13	0.04	Fruity, yogurt	0.04	1.00	1.17
Styrene	0.07	0.05	Floral, sweetness, fruity	0.01	0.20	0.23
2-Acetyl-1-pyrroline	0.0002	0.00026	Roasting, milky, popcorn	0.01	38.46	45.13
2,4-Dimethylpyrrole	0.076	0.08	Roasting, nutty	0.01	0.13	0.15
4-Methyl-2-oxo-pentanoic acid methyl ester	—	0.06	Fruity, freshness, sweetness	0.08	1.33	1.56
4-Methyl-2-oxovaleric acid	—	0.12	Fruity, sweetness	0.08	0.67	0.78
2-Pyrrolidinone	—	1.07	Sweet, milky, roasting	0.12	0.11	0.13
*N*-acetylpyrrolidone	0.11	0.11	Sweetness, popcorn	0.2	1.82	2.13
2-Ethyl-3-methyl-pyrazine	0.00035	0.00176	Roasting, nutty	0.15	85.23	100.00
Myristic acid	1.00	1.00	Milky, coconut, creamy	0.04	0.04	0.05

**Figure 6:**
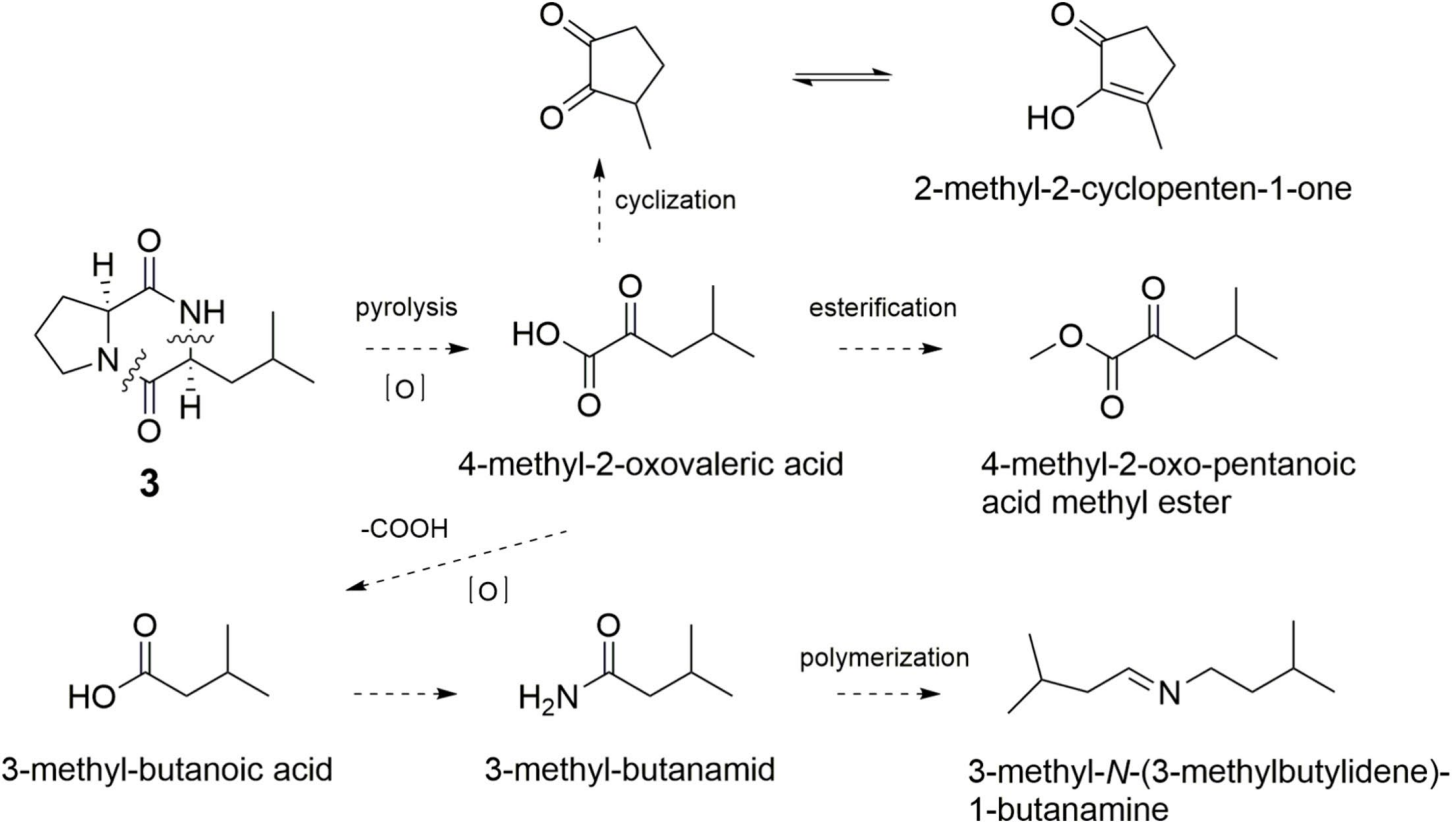
Possible formation pathway of acid and amide flavor compounds through pyrolysis of **3**.

As shown in Figure 6, the simultaneously broken of 1,2-amide bond and 8,9-C–N bond of **3** would bring an intermediate, which was further oxidized to form 4-methyl-2-oxovaleric acid and esterified to yield 4-methyl-2-oxovaleric acid methyl ester. The cyclization of 4-methyl-2-oxovaleric acid gave 2-methyl-2-cyclopenten-1-one. Decarboxylation of 4-methyl-2-oxovaleric acid afforded 3-methyl-butanoic acid, which could yield 3-methyl-butanamide and 3-methyl-*N*-(3-methylbutylidene)-1-butanamine.

**Figure 7:**
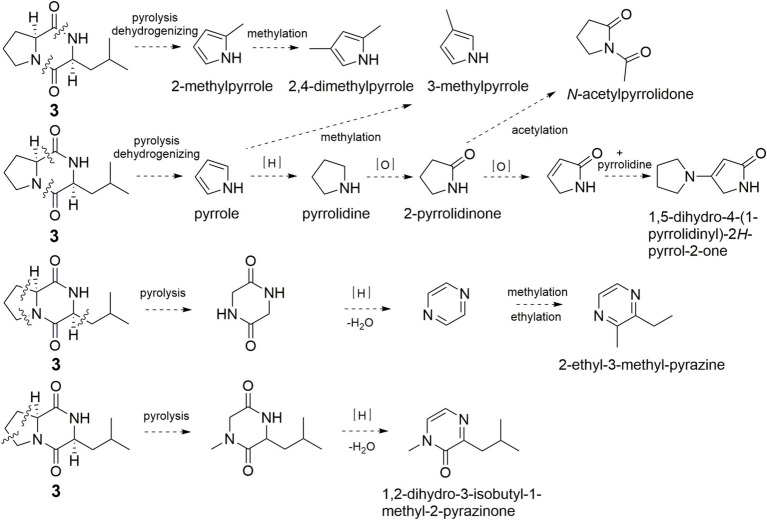
Possible formation pathway of pyrrole and pyrazine flavor compounds through pyrolysis of **3**.

It’s worth noting that quite a few pyrrole and pyrazine flavor components were found in pyrolysis products of **3** (Figure 7). The simultaneously broken of 1,2-amide bond and 6,7-C–C bond of **3** would yield pyrrole, pyrrolidine, 2-pyrrolidinone, *N*-acetylpyrrolidone, 3-methylpyrrole, and 1,5-dihydro-4-(1-pyrrolidinyl)-2*H*-pyrrol-2-one. The simultaneously broken of 1,2-amide bond and 7,8-amide bond of **3** would yield 2-methylpyrrole, which could convert to 2,4-dimethylpyrrole. Broken of 3,4-C–C bond, 6,7-C–C bond, and 9,10-C–C bond of **1** would generate pyrazine and 2-ethyl-3-methyl-pyrazine. 1,2-Dihydro-3-isobutyl-1-methyl-2-pyrazinone was formed in the same manner.

### Flavor enhancement on cigar tobacco leaves and sensory evaluation verification

The sensory evaluation was conducted on Dexue No. 1 cigar tobacco leaves, and the results were shown in Table 7. The flavors of original tobacco leaves were poor, and a noticeable level of irritation was found. In comparison with control group, the addition of *B. velezensis* and *S. equorum* fermentation broth significantly enhanced the roasting, milky, and nutty aromas in the tobacco leaves. Furthermore, the fresh sweetness, and floral aromas were also improved, resulting in a more complex overall flavor profile. The enriched flavor of cigar is likely attributed to the pyrolysis products derived from high concentrations of cyclic dipeptides present in the fermentation broth. Using Py-GC-MS detection and ROAV analysis, the characteristic flavor compounds in the pyrolysis products of cyclic dipeptides such as **1** and **3** were disclosed mainly as 6-heptyl-5,6-dihydro-2*H*-pyran-2-one, 2,5-dimethyl-3-isopropylpyrazine, 2-ethyl-3-methyl-pyrazine, and 2-acetyl-1-pyrroline. These pyrolysis flavor components mainly exhibited roasting, milky, sweetness, and nutty aromas as previously mentioned, which was highly in accordance with the sensory evaluation results. Additionally, the improvement of floral aroma in *B. velezensis* fermentation broth addition group may also be related to the flavor compound like 1-phenylethyl acetate and phenethyl butyrate in the fermentation broth.

**Table 7:** Sensory evaluation results of control groups and fermentation broth addition groups.

Aroma (scores 0–5)	Control group	*B. velezensis* fermentation broth addition group	*S. equorum* fermentation broth addition group
Roasting	2.5 ± 0.04	3.7 ± 0.05	3.3 ± 0.03
Fresh sweetness	1.8 ± 0.06	2.4 ± 0.13	2.0 ± 0.08
Milky	2.0 ± 0.11	3.4 ± 0.21	2.9 ± 0.06
Fruity	1.0 ± 0.08	1.5 ± 0.09	3.0 ± 0.10
Floral	1.0 ± 0.06	1.8 ± 0.10	1.5 ± 0.04
Nutty	1.6 ± 0.13	2.6 ± 0.16	2.5 ± 0.13

Flavor precursor compounds have been widely used in flavor and fragrance industry due to their high stability and durability (Parker et al., 2017; Xiang et al., 2023). In this study, a new kind of flavor precursors, cyclic dipeptides, was disclosed for the first time. Cyclic dipeptides can be formed by microorganisms through non-ribosomal peptide synthetase catalysis using the abundant α-amino acids in the medium (Großkopf et al., 2023; Ogilvie and Czekster, 2023). The 2,5-diketopiperazine scaffold of cyclic dipeptides is more stable than amino acids, and pyrolysis of cyclic dipeptides yields a diverse array of unique flavor compounds, thus brings continuous and stable flavoring enhancing effects. This kind of interesting molecules could be used in flavor and food industries for further development. Additionally, the floral and fruity flavor compounds 1-phenylethyl acetate and phenethyl butyrate in the fermentation broth could be yielded through the esterfication reactions of *B. velezensis*, using acetic acid, butanoic acid, 1-phenylethyl alcohol, and 2-phenylethyl alcohol as the chemical materials. Generally, acetic acid and butanoic acid are known as the glycolysis pathway products (Wang et al., 2019), while 1-phenylethyl alcohol and 2-phenylethyl alcohol are the degradation products of phenylalanine (Huang et al., 2018).

Moreover, the fermentation broth of *B. velezensis* is developed as natural flavoring exogenous additive for cigar tobacco leaves, which also provides a sustainable and efficient flavor enhancer for flavor and fragrance industry. The fermentation broth of *B. velezensis* is cost-effective, since LB liquid medium is cheap and the usage is low (Huang et al., 2018). The fermentations of flavor-producing microorganisms are considered as eco-friendly, and hardly any waste water and gas will be generated. In addition, the yielded bacterial cells of *B. velezensis* could be used as fermentation seeds, animal feeds, or biofertilizer, which is also environmental friendly and economic sustainable (Li et al., 2024a).

## Conclusion

In this study, an endophytic bacterium *B. velezensis* exhibited the ability to metabolize flavor precursor compounds. Three cyclic dipeptide flavor precursor compounds were isolated and identified in the fermentation broth as cyclo (L-prolyl-L-valine) (**1**), cyclo (L-prolyl-L-isoleucine) (**2**), and cyclo (L-prolyl-L-leucine) (**3**). The main pyrolysis products of cyclic dipeptides **1** and **3** were revealed as milky, roasting, nutty, sweetness, and fruity flavor compounds including 6-heptyl-5,6-dihydro-2*H*-pyran-2-one, isobutyric acid, 4-methyl-2-oxo-pentanoic acid, pyrrole derivatives, and pyrazine derivatives. The formation pathway of pyrolysis was also proposed in detail. The flavor enhancing effects of cyclic dipeptide flavor precursors were verified through sensory evaluation of flavored cigar tobacco leaves. It is reported for the first time that cyclic dipeptides can be used as flavor precursors. The yielded microbial fermentation flavor is natural, efficient, and economical, which can provide new insights and references for the development and application for flavor-producing microorganisms. Cyclic dipeptides, the fermentation broth flavor, and the origin flavor-producing bacterium show the potential application values in flavor and food industries. More researches about the fermentation process optimization, application extension, and flavor and yield increase are needed.

## Data Availability

The original contributions presented in the study are included in the article/Supplementary material, further inquiries can be directed to the corresponding authors.

## References

[ref1] Ayseli M. T. Ayseli Y. İ. (2016). Flavors of the future: health benefits of flavor precursors and volatile compounds in plant foods. Trends Food Sci. Technol. 48, 69–77. doi: 10.1016/j.tifs.2015.11.005, PMID: 40115879

[ref2] Braga A. Guerreiro C. Belo I. (2018). Generation of flavors and fragrances through biotransformation and de novo synthesis. Food Bioprocess Technol. 11, 2217–2228. doi: 10.1007/s11947-018-2180-8, PMID: 40115325

[ref3] Fdhila F. Vázquez V. Sánchez J. L. Riguera R. (2003). DD-Diketopiperazines: antibiotics active against *Vibrio anguillarum* isolated from marine bacteria associated with cultures of *Pecten maximus*. J. Nat. Prod. 66, 1299–1301. doi: 10.1021/np030233e, PMID: 14575426

[ref4] Großkopf J. Plaza M. Kutta R. J. Nuernberger P. Bach T. (2023). Creating a defined chirality in amino acids and cyclic dipeptides by photochemical deracemization. Angew. Chem. Int. Ed. 62:e202313606. doi: 10.1002/anie.202313606, PMID: 37793026

[ref5] Hu W. Cai W. Zheng Z. Liu Y. Luo C. Xue F.. (2022). Study on the chemical compositions and microbial communities of cigar tobacco leaves fermented with exogenous additive. Sci. Rep. 12:19182. doi: 10.1038/s41598-022-23419-y, PMID: 36357535 PMC9649726

[ref6] Huang Z. R. Hong J. L. Xu J. X. Li L. Guo W. L. Pan Y. Y.. (2018). Exploring core functional microbiota responsible for the production of volatile flavour during the traditional brewing of Wuyi Hong Qu glutinous rice wine. Food Microbiol. 76, 487–496. doi: 10.1016/j.fm.2018.07.014, PMID: 30166178

[ref7] Jiang C. Lv J. Ji L. An H. Yang M. Huang Y.. (2024). Characterization of the key aroma compounds in cigar filler tobacco leaves from different production regions. Front. Plant Sci. 15:1476807. doi: 10.3389/fpls.2024.1476807, PMID: 39737372 PMC11683104

[ref8] Kim M. J. Cheng Z. (2024). Anti-bacterial cyclic dipeptides from *Hormonema dematioides*, an endophytic fungus of *Juniperus communis*. Nat. Prod. Res. 27, 1–6. doi: 10.1080/14786419.2024.2385697, PMID: 39082367

[ref9] Li T. Li L. Zhang D. Zhao G. Liang Y. Jia X.. (2024b). Chemical constituents and bioactivities of the essential oils of *Magnolia biondii* flower buds from three provinces in China. Flavour Fragr. J. 39, 302–311. doi: 10.1002/ffj.3787

[ref10] Li T. Liang Y. Wen W. Dong H. Fan W. Dong L.. (2024a). Sweet flavor compounds produced by the endophytic fungus *Talaromyces funiculosus*. Food Sci. Biotechnol. 34, 677–685. doi: 10.1007/s10068-024-01694-x, PMID: 39958168 PMC11822143

[ref11] Li X. Nong X. Yang J. Li M. Wang Q. Sun M.. (2024). Exploring the frontier of cyclic dipeptides: a bioinformatics approach to potential therapeutic applications in schizophrenia. Int. J. Mol. Sci. 25:11421. doi: 10.3390/ijms252111421, PMID: 39518975 PMC11546255

[ref12] Li R. Yin X. Zhang S. Yang J. Zhao M. (2021). Preparation and pyrolysis of two Amadori analogues as flavor precursors. J. Anal. Appl. Pyrolysis 160:105357. doi: 10.1016/j.jaap.2021.105357, PMID: 40115879

[ref13] Liu C. Du Y. Zheng J. Qiao Z. Luo H. Zou W. (2022). Production of caproic acid by *Rummeliibacillus suwonensis* 3B-1 isolated from the pit mud of strong-flavor baijiu. J. Biotechnol. 358, 33–40. doi: 10.1016/j.jbiotec.2022.08.017, PMID: 36049550

[ref14] Liu R. Kim A. H. Kwak M. K. Kang S. O. (2017). Proline-based cyclic dipeptides from Korean fermented vegetable kimchi and from *Leuconostoc mesenteroides* LBP-K06 have activities against multidrug-resistant bacteria. Front. Microbiol. 8:761. doi: 10.3389/fmicb.2017.00761, PMID: 28512456 PMC5411444

[ref15] Mindt M. Ferrer L. Bosch D. Cankar K. Wendisch V. F. (2023). *De novo* tryptophanase-based indole production by metabolically engineered *Corynebacterium glutamicum*. Appl. Microbiol. Biotechnol. 107, 1621–1634. doi: 10.1007/s00253-023-12397-4, PMID: 36786915 PMC10006044

[ref16] Niu Y. Yao Z. Xiao Z. Zhu G. Zhu J. Chen J. (2018). Sensory evaluation of the synergism among ester odorants in light aroma-type liquor by odor threshold, aroma intensity and flash GC electronic nose. Food Res. Int. 113, 102–114. doi: 10.1016/j.foodres.2018.01.018, PMID: 30195503

[ref17] Ogilvie C. E. Czekster C. M. (2023). Cyclic dipeptides and the human microbiome: opportunities and challenges. Bioorg. Med. Chem. 90:117372. doi: 10.1016/j.bmc.2023.117372, PMID: 37343497

[ref18] Parker M. Capone D. L. Francis I. L. Herderich M. J. (2017). Aroma precursors in grapes and wine: flavor release during wine production and consumption. J. Agric. Food Chem. 66, 2281–2286. doi: 10.1021/acs.jafc.6b05255, PMID: 28220693

[ref19] Pérez-Picaso L. Olivo H. F. Argotte-Ramos R. Rodríguez-Gutiérrez M. Rios M. Y. (2012). Linear and cyclic dipeptides with antimalarial activity. Bioorg. Med. Chem. Lett. 22, 7048–7051. doi: 10.1016/j.bmcl.2012.09.094, PMID: 23084276

[ref20] Qian H. Kang X. Hu J. Zhang D. Liang Z. Meng F.. (2020). Reversing a model of Parkinson’s disease with *in situ* converted nigral neurons. Nature 582, 550–556. doi: 10.1038/s41586-020-2388-4, PMID: 32581380 PMC7521455

[ref21] Schmidtz F. J. Vanderah D. J. Hollenbeak K. H. Enwall C. E. Gopichand Y. SenGupta P. K.. (1983). Metabolites from the marine sponge *Tedania ignis*: a new atisanediol and several known diketopiperazines. J. Org. Chem. 48, 3941–3945. doi: 10.1021/jo00170a011, PMID: 40104789

[ref22] Wang G. Yu Y. Wang Y. Z. Wang J. J. Guan R. Sun F.. (2019). Role of SCFAs in gut microbiome and glycolysis for colorectal cancer therapy. J. Cell. Physiol. 234, 17023–17049. doi: 10.1002/jcp.28436, PMID: 30888065

[ref23] Xiang Q. Xia Y. Chen L. Chen M. Wang D. Zhong F. (2023). Flavor precursors and flavor compounds in Cheddar-flavored enzyme-modified cheese due to pre-enzymolysis combined with lactic acid bacteria fermentation. Food Biosci. 53:102698. doi: 10.1016/j.fbio.2023.102698

[ref24] Yan Y. Sun L. Xing X. Wu H. Lu X. Zhang W.. (2022). Microbial succession and exploration of higher alcohols-producing core bacteria in northern Huangjiu fermentation. AMB Express 12:79. doi: 10.1186/s13568-022-01418-6, PMID: 35716260 PMC9206695

[ref25] Yuan S. Jin Z. Ali A. Wang C. Liu J. (2022). Caproic acid-producing bacteria in Chinese Baijiu brewing. Front. Microbiol. 13:883142. doi: 10.3389/fmicb.2022.883142, PMID: 35602080 PMC9114508

[ref26] Zang J. Yu D. Zhang P. Xu Y. Xia W. (2022). The key enzymes and flavor precursors involved in formation of characteristic flavor compounds of low-salt fermented common carp (*Cyprinus carpio* L.). LWT 154:112806. doi: 10.1016/j.lwt.2021.112806, PMID: 40115879

