# Supplementary material for: Cyclic dipeptides from endophytic bacterium Bacillus velezensis as potential flavor precursors
Source: Front Microbiol. 2025 Apr 1;16:1565502. doi: 10.3389/fmicb.2025.1565502 (PMC11996796; doi:10.3389/fmicb.2025.1565502)
Supplement: Supplementary file 1 [file Data_Sheet_1.docx]

**Table of Contents**

**TABLE S1** GC-MS analysis of main aroma components in fermentation broth of *S. equorum*.......................................................................................................................S1

**TABLE S2** ^1^H (500M Hz) and ^13^C NMR (125 MHz) NMR data of **1**-**3** in CD_3_OD........................................................................................................................S2

**For compound 1**

**Figure S1.** ESIMS spectrum of **1**……..................................…………....….…........S3

**Figure S2.** ^1^H NMR spectrum (500 MHz, CD_3_OD) of **1**….......................................S3

**Figure S3.** ^13^C NMR spectrum (500 MHz, CD_3_OD) of **1**…......................................S4

**For compound 2**

**Figure S4.** ESIMS spectrum of **2**……..................................…………....….…........S4

**Figure S5.** ^1^H NMR spectrum (500 MHz, CD_3_OD) of **2**….......................................S5

**Figure S6.** ^13^C NMR spectrum (500 MHz, CD_3_OD) of **3**…......................................S5

**For compound 3**

**Figure S7.** ESIMS spectrum of **3**……..................................…………....….…........S6

**Figure S8.** ^1^H NMR spectrum (500 MHz, CD_3_OD) of **2**….......................................S6

**Figure S9.** ^13^C NMR spectrum (500 MHz, CD_3_OD) of **3**…......................................S7

**TABLE S1 GC-MS analysis of main aroma components in fermentation broth of *S. equorum.***

| No. | *t*_R_(min) | *RI*/(cal./ref.) | Qualitative | Flavor compounds | Concentration (μg/mL) |
| --- | --- | --- | --- | --- | --- |
| 1 | 10.72 | 876/875 | RI, MS, S | 3-Methylbutanoic acid | 2.69 |
| 2 | 11.37 | 896/897 | RI, MS, S | 2-Methylbutanoic acid ethyl ester | 6.08 |
| 3 | 13.91 | 1061/1060 | RI, MS, S | Phenethyl alcohol | 0.73 |
| 4 | 15.78 | 1174/1173 | RI, MS, S | Ethyl benzoate | 0.57 |
| 5 | 17.83 | 1200/1200 | RI, MS, S | Dodecane | 0.39 |
| 6 | 19.33 | ― | MS, S | Ethyl 3-hydroxybutyrate | 2.98 |
| 7 | 22.69 | 1364/1366 | RI, MS, S | 2,6-Dimethoxyphenol | 1.03 |
| 8 | 26.78 | 1584/1585 | RI, MS, S | Diethyl phthalate | 0.52 |
| 9 | 31.73 | 1685/1684 | RI, MS | (*E*)-14-Hexadecenal | 0.10 |
| 10 | 32.91 | 1796/1795 | RI, MS, S | Hexahydro-3-methylpyrrolo[1,2-a]pyrazine-1,4-dione | 4.32 |
| 11 | 34.47 | ― | MS | Cyclo(L-prolyl-L-glycine) | 19.31 |
| 12 | 36.21 | 1801/1803 | RI, MS, S | Cyclo(L-prolyl-L-valine) | 7.80 |
| 13 | 37.20 | 1887/1885 | RI, MS, S | 1-Nonadecene | 0.13 |
| 14 | 38.26 | ― | MS | Hexahydro-3-(1-methylpropyl)pyrrolo[1,2-a]pyrazine-1,4-dione | 1.78 |
| 15 | 38.91 | 1907/1908 | RI, MS, S | Cyclo(L-prolyl-L-leucine) | 14.41 |
| 16 | 42.12 | 1991/1994 | RI, MS, S | 1-Eicosene | 0.59 |
| 17 | 43.86 | ― | MS, S | 3,6-Bis(2-methylpropyl)-2,5-piperazinedione | 11.91 |
| 18 | 48.69 | ― | MS, S | cyclo(L-prolyl-L-phenylalanine) | 8.10 |
| 19 | 49.91 | 2000/2000 | RI, MS, S | Eicosane | 0.13 |
| 20 | 51.62 | 2373/2375 | RI, MS, S | (Z)-9-Octadecenamide | 0.18 |
| 21 | 52.47 | 2387/2390 | RI, MS, S | 1-Tetracosene | 0.08 |
| 22 | 52.93 | 2551/2550 | RI, MS, S | Bis(2-ethylhexyl) phthalate | 20.02 |
| 23 | 54.86 | 2624/2625 | RI, MS, S | (*Z*)-13-Docosenamide | 3.28 |

**TABLE S2 ^1^H (500M Hz) and ^13^C NMR (125 MHz) NMR data of 1-3 in CD_3_OD.**

| No. | **1** | |  | **2** | |  | **3** | |
| --- | --- | --- | --- | --- | --- | --- | --- | --- |
|  | *δ*_H_ (mult, *J* in Hz) | *δ*_C_ |  | *δ*_H_ (mult, *J* in Hz) | *δ*_C_ |  | *δ*_H_ (mult, *J* in Hz) | *δ*_C_ |
| 1 |  | 167.7 |  |  | 167.7 |  |  | 169.1 |
| 3a | 3.56, m | 46.3 |  | 3.55, m | 46.3 |  | 3.51, m | 46.6 |
| 3b | 3.50, m |  |  | 3.50, m |  |  | 3.51, m |  |
| 4a | 2.02, m | 23.4 |  | 2.02, m | 23.4 |  | 2.02, m | 23.8 |
| 4b | 1.95, m |  |  | 1.96, m |  |  |  |  |
| 5a | 2.48, m | 30.1 |  | 2.32, m | 29.7 |  | 2.30, m | 29.2 |
| 5b | 2.32, m |  |  | 1.96, m |  |  | 1.96, m |  |
| 6 | 4.20, t, 7.3 | 61.7 |  | 4.20, m | 61.5 |  | 4.26, t, 7.3 | 60.4 |
| 7 |  | 173.0 |  |  | 172.6 |  |  | 172.9 |
| 9 | 4.03, s | 60.2 |  | 4.03, s | 60.1 |  | 4.13, br s | 54.8 |
| 10 | 1.95, m | 29.4 |  | 2.16, m | 37.2 |  | 1.90, m | 39.6 |
| 11a | 1.09, d, 7.3 | 19.0 |  | 1.43, m | 23.3 |  | 1.51, m | 25.9 |
| 11b |  |  |  | 1.32, m |  |  |  |  |
| 12 | 0.94, d, 6.9 | 16.8 |  | 0.93, t, 7.4 | 12.7 |  | 0.97, d, 6.0 | 23.4 |
| 13 |  |  |  | 1.07, d, 7.1 | 15.7 |  | 0.95, d, 6.0 | 22.4 |


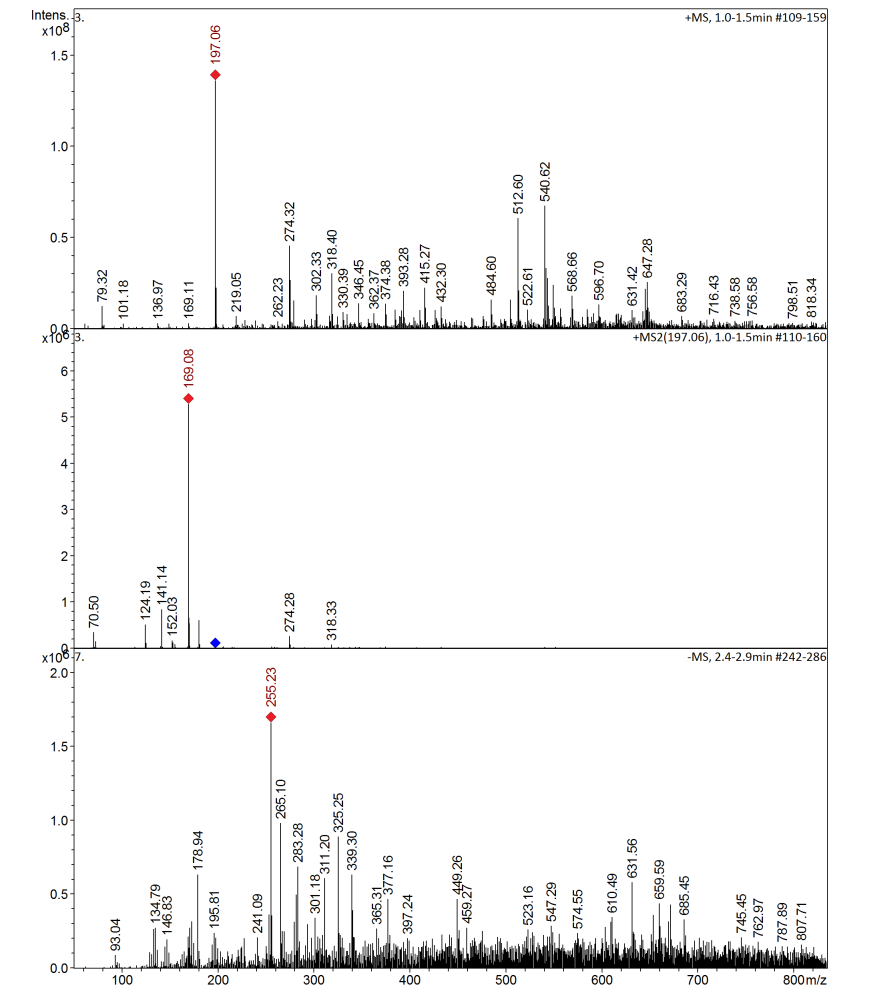


**Figure S1.** ESIMS spectrum of **1**


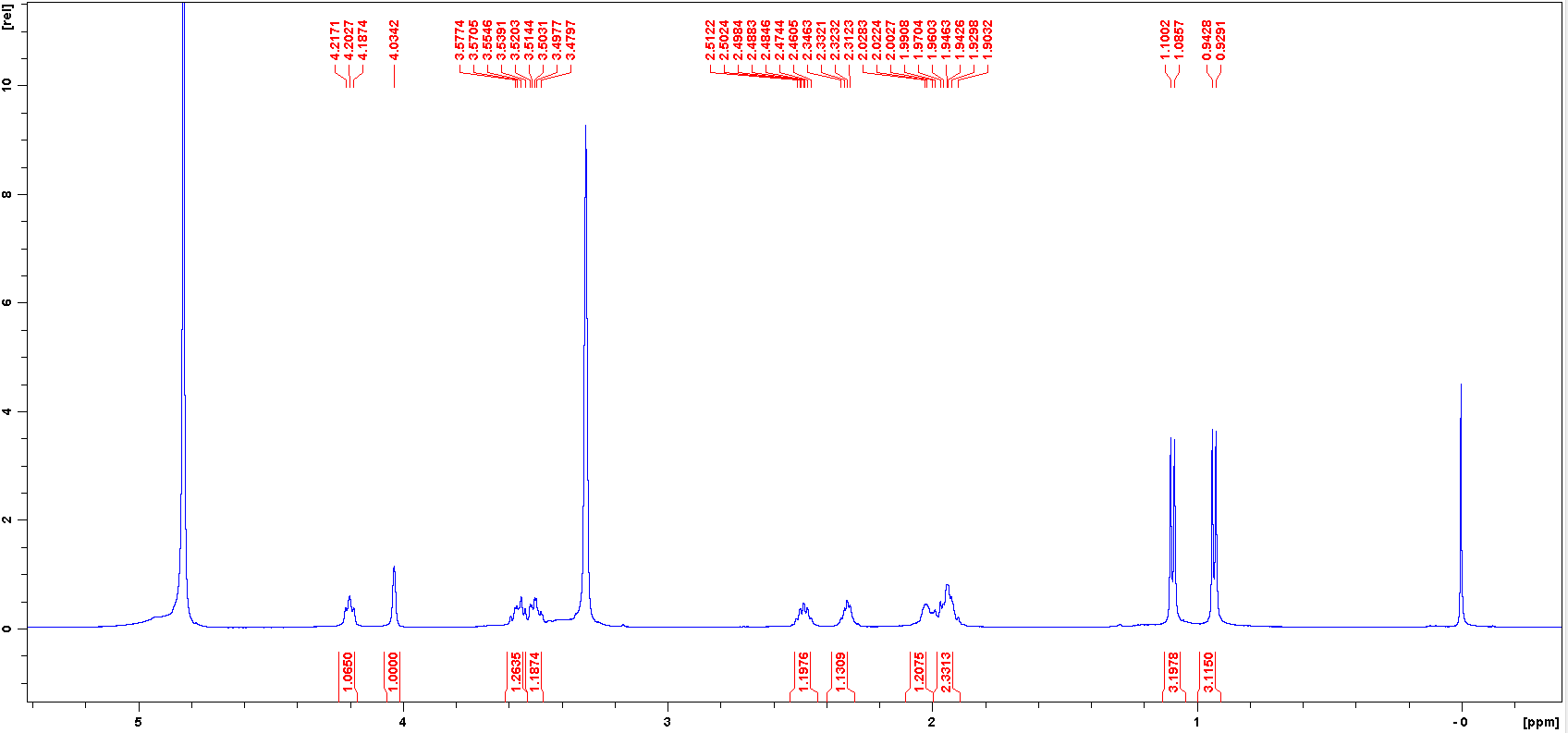


**Figure S2.** ^1^H NMR spectrum (500 MHz, CD_3_OD) of **1**


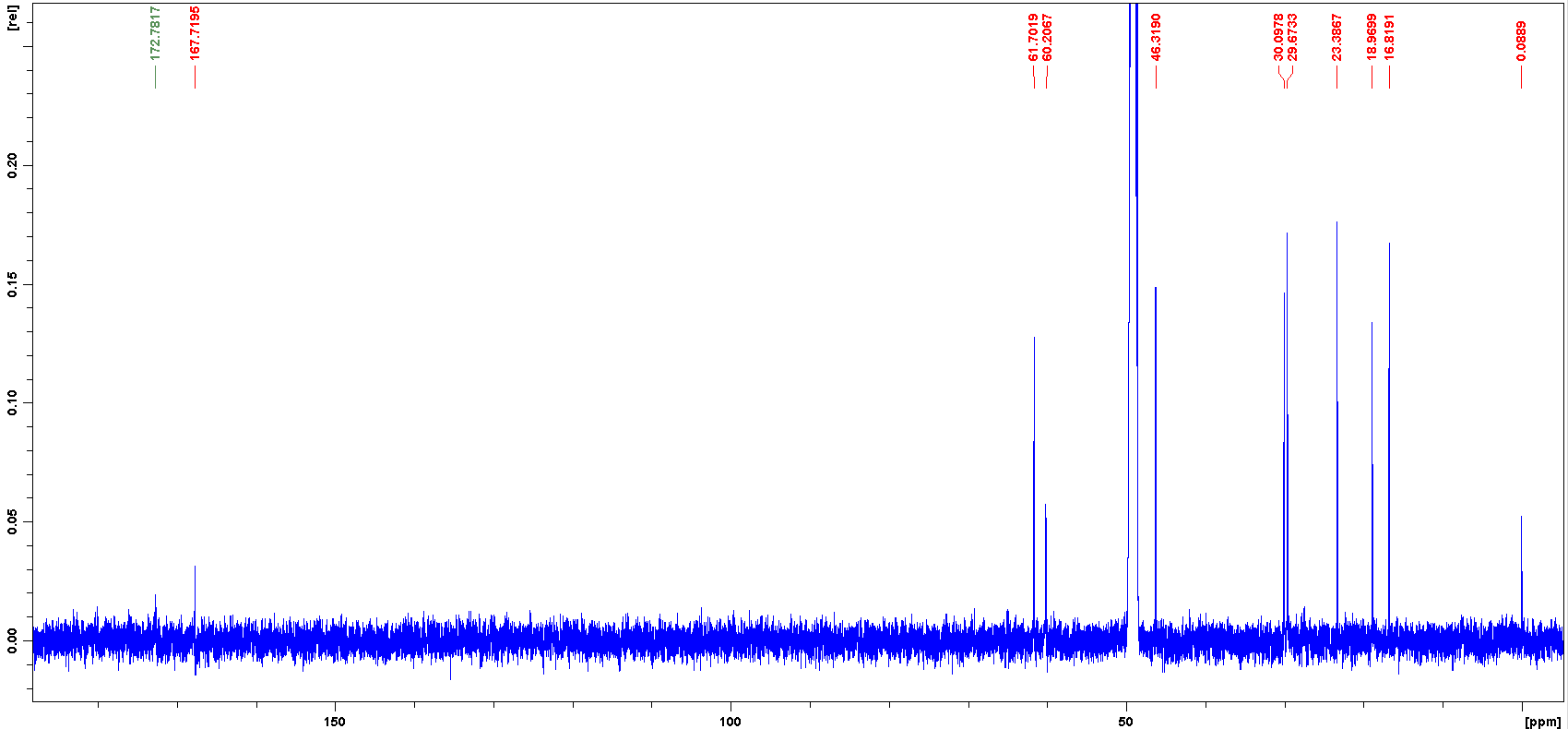


**Figure S3.** ^13^C NMR spectrum (500 MHz, CD_3_OD) of **1**


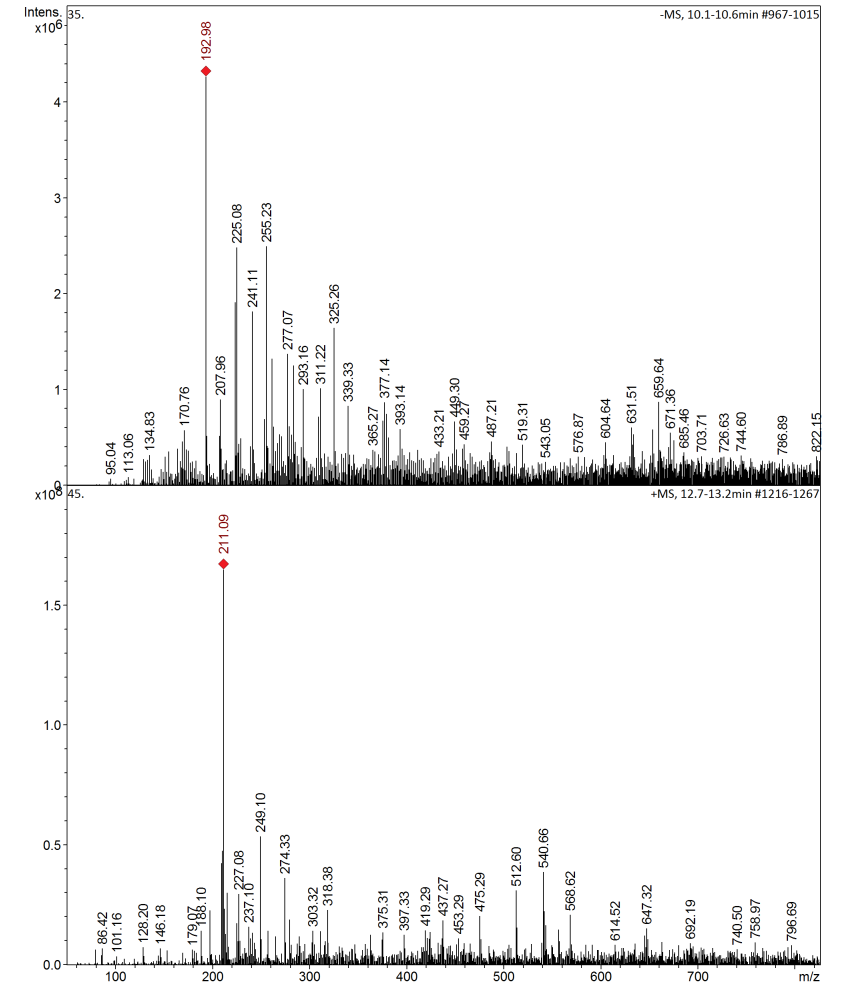


**Figure S4.** ESIMS spectrum of **2**


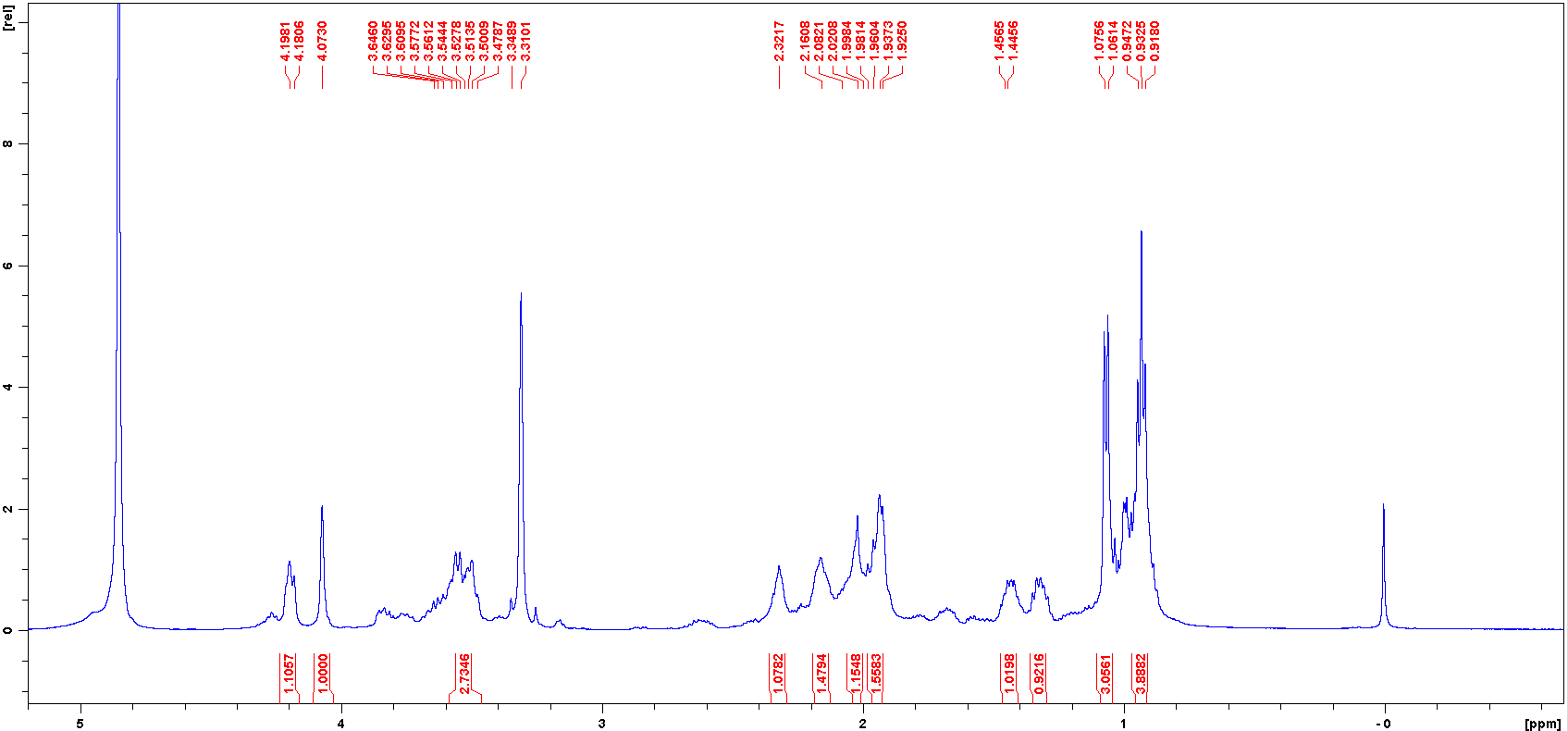


**Figure S5.** ^1^H NMR spectrum (500 MHz, CD_3_OD) of **2**


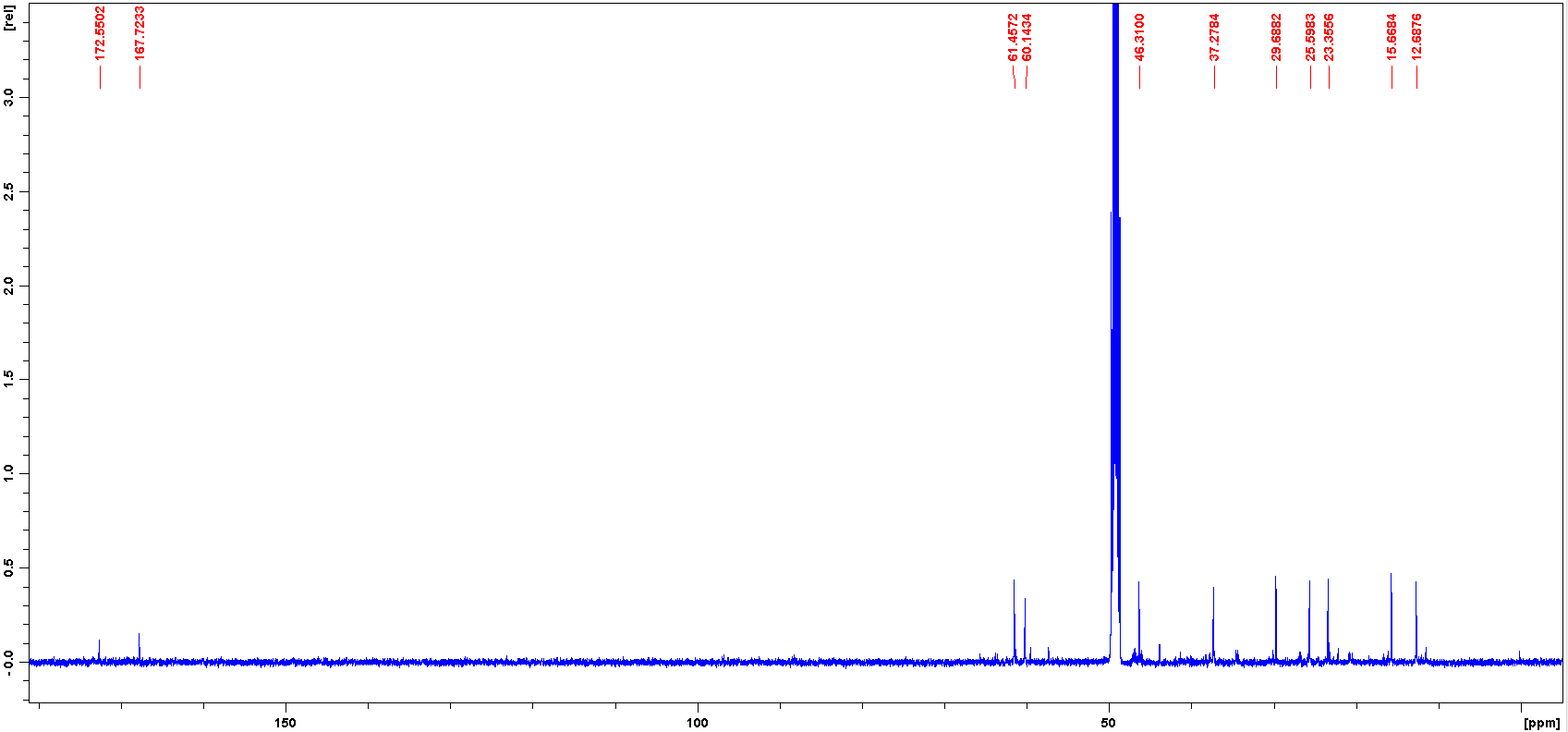


**Figure S6.** ^13^C NMR spectrum (500 MHz, CD_3_OD) of **2**


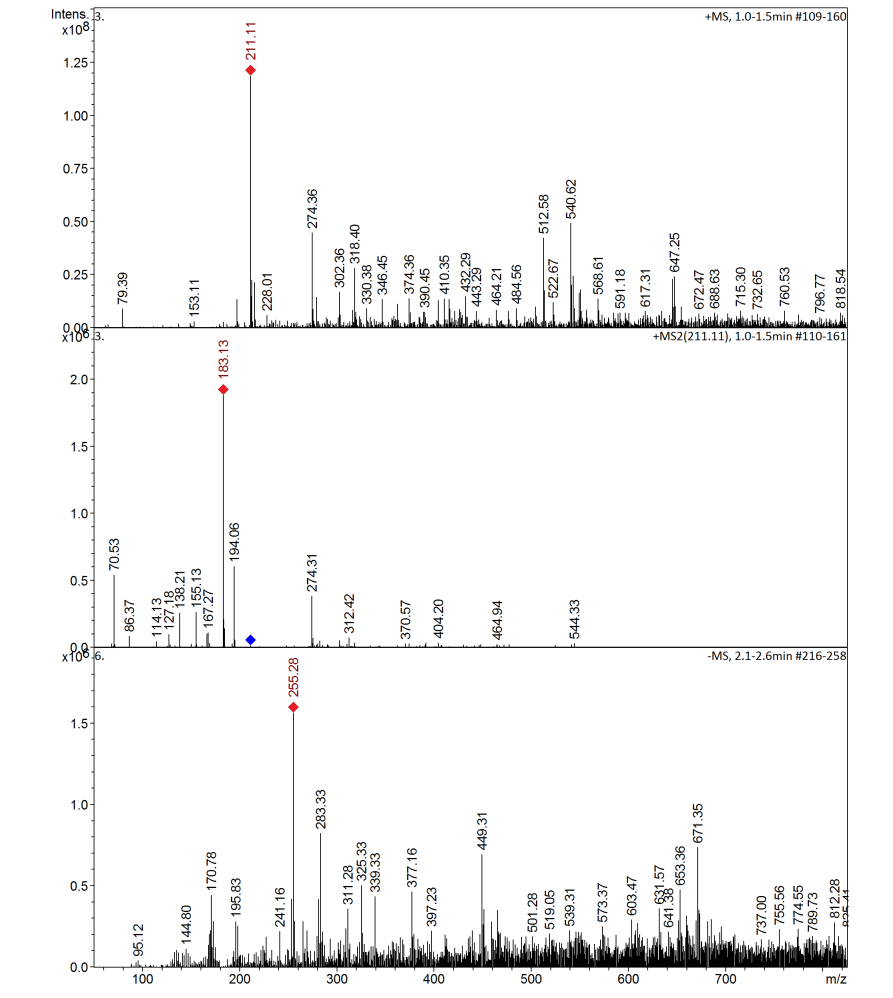


**Figure S7.** ESIMS spectrum of **3**


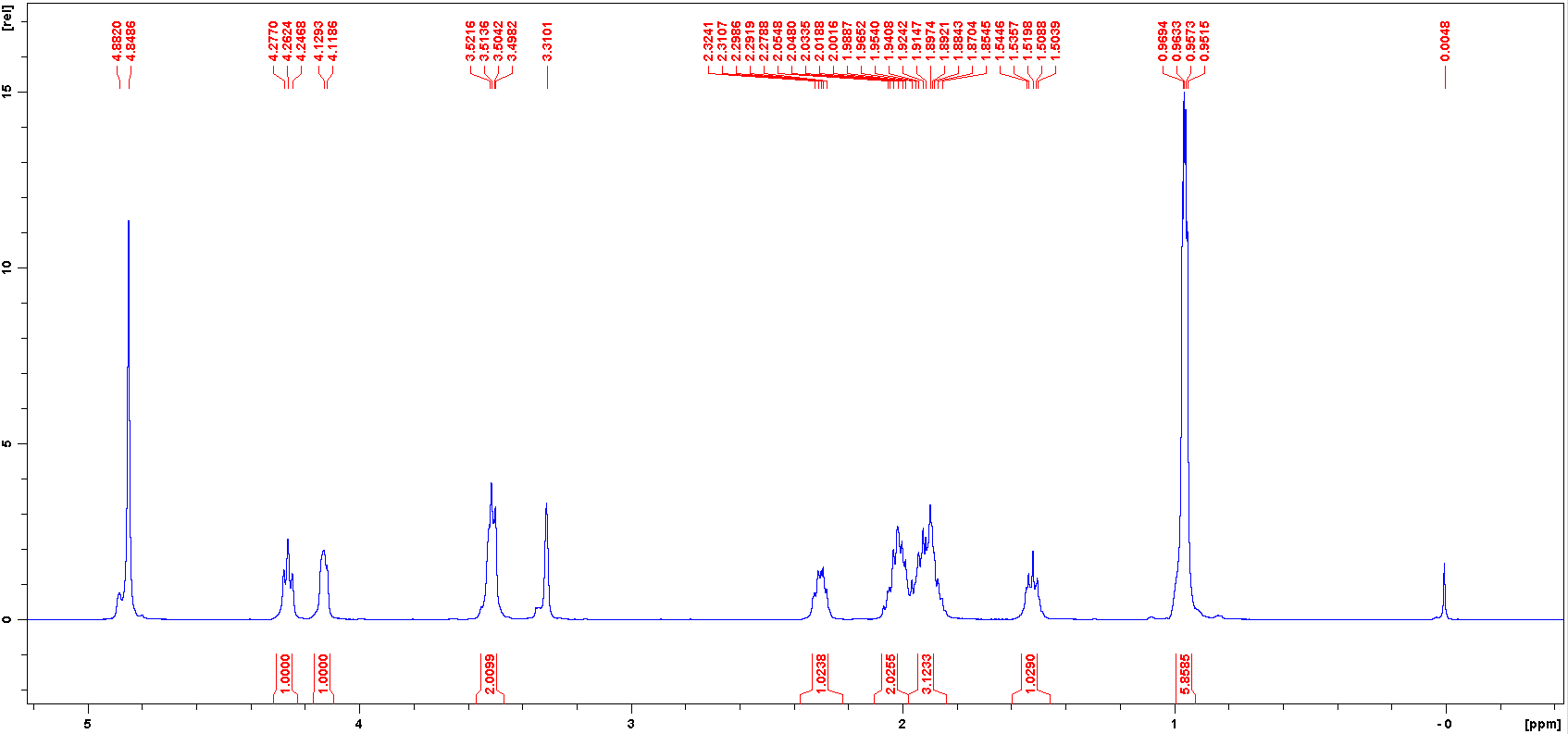


**Figure S8.** ^1^H NMR spectrum (500 MHz, CD_3_OD) of **3**


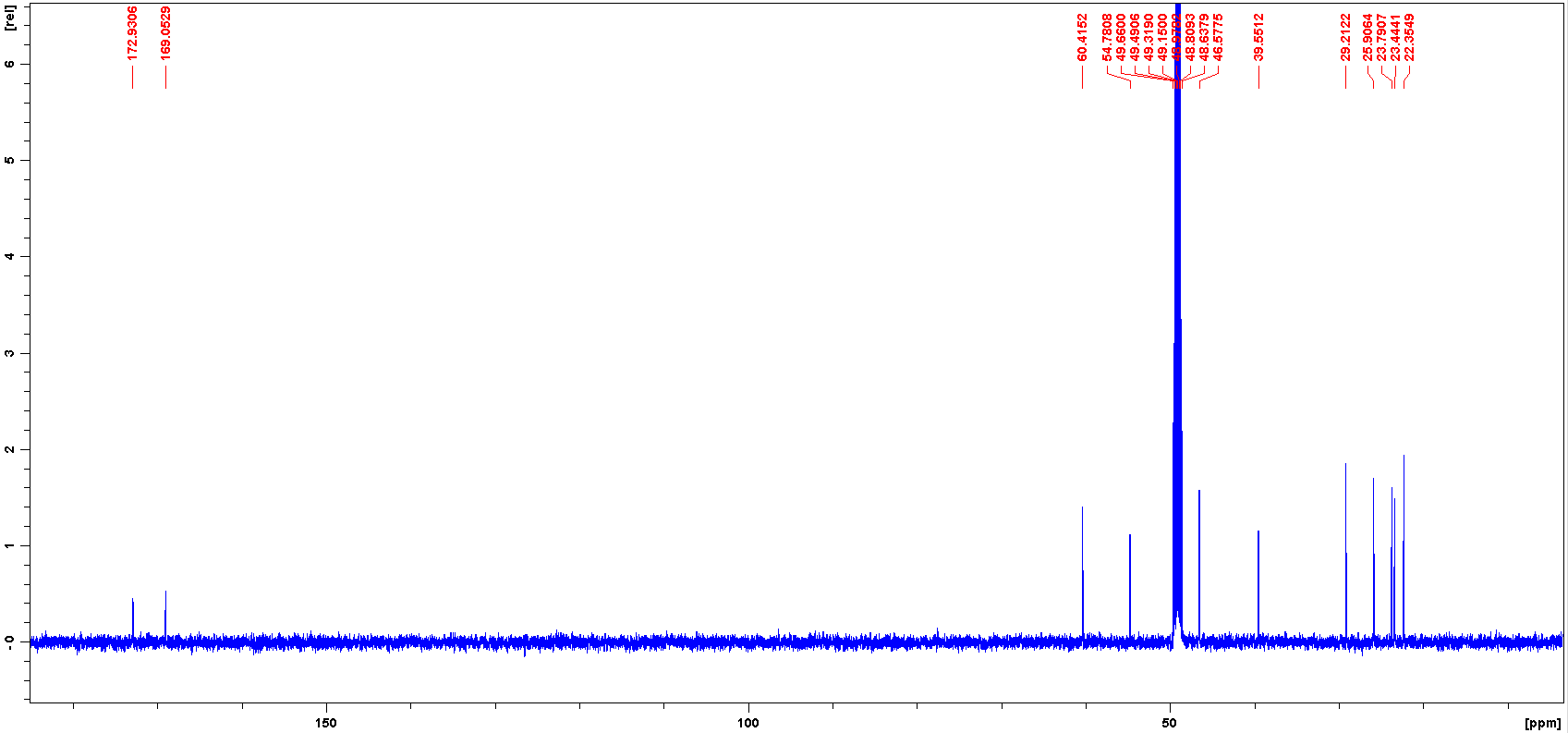


**Figure S9.** ^13^C NMR spectrum (500 MHz, CD_3_OD) of **3**
